# Metabolomic Markers in Attention-Deficit/Hyperactivity Disorder (ADHD) among Children and Adolescents—A Systematic Review

**DOI:** 10.3390/ijms25084385

**Published:** 2024-04-16

**Authors:** Elena Predescu, Tudor Vaidean, Andreea-Marlena Rapciuc, Roxana Sipos

**Affiliations:** 1 Department of Neuroscience, Psychiatry and Pediatric Psychiatry, “Iuliu Hatieganu” University of Medicine and Pharmacy, 57 Republicii Street, 400489 Cluj-Napoca, Romania; predescu.elena@umfcluj.ro; 2 Clinic of Pediatric Psychiatry and Addiction, Clinical Emergency Hospital for Children, 57 Republicii Street, 400489 Cluj-Napoca, Romania; tudor.vaidean@elearn.umfcluj.ro; 3 Clinical Department of Nephrology, County Emergency Clinical Hospital Cluj, 3-5 Clinicilor Street, 400006 Cluj-Napoca, Romania; andreea.marl.rapciuc@elearn.umfcluj.ro

**Keywords:** ADHD, metabolomic, biomarkers, amino acid metabolism, neurotransmitters, kynurenine pathway, melatonin, oxidative stress

## Abstract

Attention-Deficit/Hyperactivity Disorder (ADHD), characterized by clinical diversity, poses diagnostic challenges often reliant on subjective assessments. Metabolomics presents an objective approach, seeking biomarkers for precise diagnosis and targeted interventions. This review synthesizes existing metabolomic insights into ADHD, aiming to reveal biological mechanisms and diagnostic potentials. A thorough PubMed and Web of Knowledge search identified studies exploring blood/urine metabolites in ADHD-diagnosed or psychometrically assessed children and adolescents. Synthesis revealed intricate links between ADHD and altered amino acid metabolism, neurotransmitter dysregulation (especially dopamine and serotonin), oxidative stress, and the kynurenine pathway impacting neurotransmitter homeostasis. Sleep disturbance markers, notably in melatonin metabolism, and stress-induced kynurenine pathway activation emerged. Distinct metabolic signatures, notably in the kynurenine pathway, show promise as potential diagnostic markers. Despite limitations like participant heterogeneity, this review underscores the significance of integrated therapeutic approaches targeting amino acid metabolism, neurotransmitters, and stress pathways. While guiding future research, this overview of the metabolomic findings in ADHD suggests directions for precision diagnostics and personalized ADHD interventions.

## 1. Introduction

In the dynamic field of pediatric psychiatry, understanding the intricate biological basics of Attention-Deficit/Hyperactivity Disorder (ADHD) is essential for advancing diagnostic precision and treatment efficacy. This systematic review aims to explore and synthesize existing knowledge on key metabolomic markers found in the blood and urine which are associated with ADHD in children and adolescents.

ADHD stands as one of the most prevalent neurodevelopmental disorders, affecting approximately 5–10% of children and adolescents worldwide [1]. This condition is characterized by persistent patterns of inattention, hyperactivity, and impulsivity, leading to significant challenges in various aspects of life, including academic performance, social interactions, and overall daily functioning [2]. Its impact extends beyond the individual, affecting families [3], teachers, and the broader societal structure [4]. The clinical presentation of ADHD is notably intricate, with variations in symptomatology contributing to its classification into three subtypes: predominantly inattentive, predominantly hyperactive–impulsive, and combined [5,6]. Treatment of ADHD is multifaceted, often involving a combination of behavioral interventions, psychoeducation, and pharmacotherapy. While stimulant medications, such as methylphenidate and amphetamines, have demonstrated efficacy in managing symptoms [7], non-stimulant options, including atomoxetine and guanfacine, offer alternatives, particularly in cases of intolerance or insufficient response to stimulants [8]. The individualized nature of treatment underscores the importance of considering comorbid conditions and tailoring interventions to address specific symptom profiles. Moreover, ADHD frequently coexists with other mental health conditions, adding complexity to its clinical management [9]. Young children may not exhibit distinct symptom profiles, and the symptoms of ADHD may overlap with those of other developmental disorders, making it challenging to differentiate between different clinical scenarios.

The understanding of ADHD has evolved significantly over the years, transitioning beyond a purely behavioral point of view, to encompass a deeper exploration of the biological factors and the intricate interplay between neurobiology, genetics, and environmental influences in shaping the ADHD phenotype [5,10]. Traditional diagnostic practices heavily rely on behavioral observations, subjective assessments, and symptom checklists, which, while valuable, may lack the specificity and precision required for accurate and early identification of ADHD [11,12]. One of the primary challenges in ADHD diagnosis lies in its heterogeneity, with individuals manifesting a spectrum of symptoms and variations in their severity and often presenting with comorbid conditions, further complicating the diagnostic process and potentially leading to misdiagnosis or delayed intervention [5]. However, as advancements in neuroscience and genetics have unfolded, there has been a notable paradigm shift in the conceptualization of ADHD [13]. The emergence of neuroimaging studies, molecular genetics, and other advanced methodologies has enabled a deeper exploration of the neural circuits and neurotransmitter systems implicated in ADHD [14,15,16]. Structural and functional brain imaging studies have identified differences in brain regions associated with attention, impulse control, and executive functions among individuals with ADHD. Moreover, the recognition of the heritability of ADHD has driven investigations into genetic factors contributing to its onset and persistence [17,18,19]. The recognition of ADHD as a multifaceted disorder with both genetic and environmental influences has prompted an ongoing exploration of its underlying biological mechanisms [10,20].

Metabolomics, the systematic study of small molecules or metabolites in biological systems, offers a unique view into the metabolic signatures associated with various physiological and pathological states [21]. Metabolite biosignatures detected in human biofluids, bridging the gap between genotype, environment, and phenotype, serve as compelling biomarkers for clinical diagnosis, prognosis, and the classification of diseases [22,23]. Often deemed as immediate indicators of diseases due to their close connection with underlying pathogenic mechanisms, metabolites play a pivotal role in clinical diagnostics [24]. However, the traditional timeline for translating the discovery of disease markers from initial identification to routine clinical implementation usually spans a decade or more. The advancements in metabolomics technologies provide a promising opening for a more rapid and efficient discovery and validation of metabolic indicators [25]. Metabolomic markers have the potential to serve as objective and quantitative indicators of the physiological alterations associated with ADHD [26], distinguishing between comorbidities and elucidating pathophysiology. The application of metabolomics in ADHD research holds promise for several reasons: metabolomic markers can provide objective and quantifiable measures, reducing the subjectivity associated with traditional diagnostic methods; it may enable the identification of ADHD at an early stage, facilitating timely interventions and personalized treatment strategies [25]; and it has the potential to contribute to the emerging field of precision medicine and allows for a comprehensive exploration of the intricate biological processes contributing to ADHD [27], complementing the information obtained from behavioral assessments.

The existing literature on ADHD and metabolomics is dispersed and lacks a comprehensive synthesis. Previous studies have reported disparate findings or utilized varied methodologies. The field of metabolomics in ADHD is dynamic, with studies sometimes reporting conflicting results. By describing consistent metabolomic markers associated with ADHD, this systematic review aims to contribute to diagnostic tool improvement, enhancing diagnostic precision and aiding in early intervention strategies. Understanding the metabolic basis of ADHD can potentially lead to the development of targeted therapeutic interventions.

The primary objectives of this systematic review are: (1) To systematically identify and analyze the principal metabolites whose blood or urinary levels show potential for utilization in distinguishing individuals with ADHD among children and adolescents; (2) to explore and assess the correlation between these identified metabolites and various clinical aspects of ADHD, including manifestations, subtypes, as well as performance on diverse psychometric tests, and additionally, to investigate potential correlations with other aspects such as neuroimaging findings; (3) to synthetize the theoretical foundations underlying the discovered metabolomic results. This involves placing a particular emphasis on the identification of intricacies within different metabolic pathways implicated in ADHD, providing a comprehensive understanding of the theoretical framework supporting the observed results.

## 2. Methods

Our study followed the reporting guidelines outlined in The Preferred Reporting Items for Systematic Reviews and Meta-Analyses (PRISMA) [28]. The primary focus of our search was to address the question: Which specific metabolites, measured in blood or urine, display promising potential in distinguishing individuals with ADHD among children and adolescents?

*Information Sources:* To identify relevant studies, we employed a search string designed as ((metabolom*) OR (metabolit*) OR (omics) OR (plasma) OR (serum) OR (urin*)) AND ((adhd) OR (hyperkine*) OR (attention defic*)) AND ((children) OR (adolescent*)). This string was adapted to meet platform requirements for identifying key terms in the titles and/or abstracts of articles indexed in PubMed and Web of Knowledge, as detailed in Supplementary File S1.

*Eligibility Criteria:* Our inclusion criteria comprised studies involving children and/or adolescents (age range: 1–18 years, with two studies extending to 19 years) professionally diagnosed with ADHD or assessed using validated psychometric instruments. Collection of blood and/or urine specimens from participants, enabling the quantification of intermediary or final metabolites within diverse biochemical processes, followed by comparative analyses between these values with either a control group or established reference laboratory values, was essential. Exclusion criteria comprised studies identifying metabolites of exogenous toxic compounds and intervention studies, unless baseline-level comparisons with a reference value were provided. Certain compounds, such as microelements, vitamins, sex hormones, and their metabolites, were deliberately omitted due to the heterogeneous nature of the literature on these subjects. We excluded commentaries, editorials, protocols, papers written in languages other than English, theses, dissertations, systematic reviews and meta-analyses, and studies conducted on animal models.

**Figure 1:**
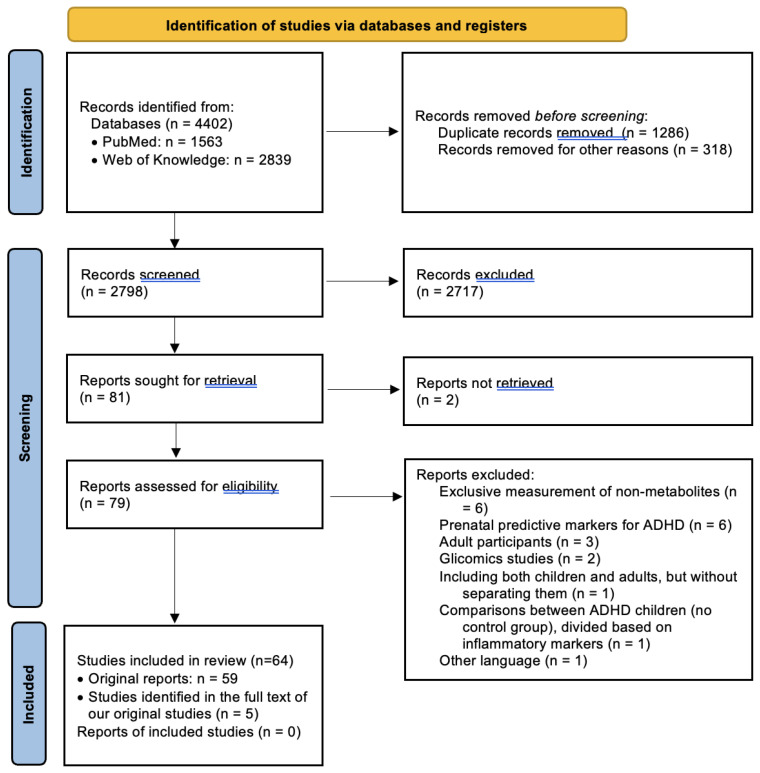
PRISMA flow diagram for systematic reviews [28].

*Study Selection:* The last search in both databases was conducted unilaterally by a single reviewer in February 2024. Following the initial search process, we identified 4402 results, of which 1287 duplicates were eliminated. Subsequently, after excluding materials other than articles (such as conference abstracts and correspondences), systematic reviews, and meta-analyses, 2797 studies remained for title and abstract screening. The same reviewer screened the studies based on inclusion and exclusion criteria, initially reviewing titles and abstracts and subsequently examining full texts. At this stage, 80 clinically relevant studies were identified for full-text review. Sequentially, 21 of these were excluded for various reasons: 6 studies which identified potentially predictive prenatal markers for ADHD [29,30,31,32,33,34]; 3 which comprised exclusively adult samples [35,36,37], as well as 1 study that included both children and adults without separately presenting differences between healthy children and those with ADHD [38]; 5 studies that did not measure the metabolites themselves [39,40,41,42,43]; 2 that were based on glycomic markers [44,45]; 2 for which the full text could not be obtained [46,47]; 1 study which compared ADHD children divided on the basis of inflammatory marker levels, with no control group [48]; and 1 study which had the full text available only in Catalan [49]. Additionally, through a thorough examination of these articles, as well as the identified reviews and meta-analyses, we identified 5 additional resources suitable for inclusion in the present review, resulting in a final number of 64 included studies. The entire process is illustrated in Figure 1.

*Quality Assessment and Interrater Reliability:* The quality of each included study was independently assessed by two of the authors using an adapted version of the Newcastle–Ottawa Scale for cross-sectional studies. Although such adaptations have been utilized in various works, it demonstrated a moderate inter-rater agreement (determined by dividing the number of agreements by the total number of scores) in a recent study [50]. The evaluation of study quality followed a star-based system, assigning scores on a scale of 0 to 8. Discrepancies were addressed through consensus discussion. The results of this assessment are presented in Supplementary Table S1.

*Data Extraction:* From each study, the following information was extracted: participant sample characteristics, including classification and group size, age ranges, gender distribution, psychiatric status, and medication taken by participants. Additionally, the techniques used to measure the markers of interest, the metabolites that were compared, and any significant correlations with clinically relevant aspects were identified. Given the heterogeneity of the included studies in all aspects (methodology, variables of interest, analysis and reporting methods), effect size indicators were not used. Instead, all metabolomic markers with significant differences in individuals with ADHD were synthesized, attempting grouping based on the main metabolic processes involved. However, this was not always possible, as some studies reported changes in the levels of several compounds which were representative of different metabolic pathways. Additionally, untargeted metabolomic studies were grouped separately due to the impossibility of categorizing the variety of identified markers into a single category from the previous ones.

## 3. Results and Discussion

The results of the included studies on metabolomic markers in ADHD reveal significant variations across five major metabolic processes. Notable findings suggest heightened oxidative stress, disruptions in lipid balance crucial for the nervous system, alterations in multiple amino acids (with tryptophan playing a pivotal role in various emergent processes), dysregulation of the kynurenine pathway, and changes in neurotransmitter metabolism. Despite attempts to categorize these processes, their interconnections are intricate, emphasizing their operation within a complex framework. Recent untargeted metabolomic studies have identified numerous markers not previously reported in the literature, enhancing our understanding of the underlying pathophysiological processes in ADHD. Table 1 presents the primary characteristics and outcomes of the studies included in this systematic review, categorized based on the main targeted metabolic processes. In addition, Supplementary Table S2 presents a simplified and systematized version of the main results from these studies.

The analysis of omega-3 and omega-6 fatty acids in different sample types (plasma, whole blood, and erythrocyte membrane) revealed varying trends across studies. Particularly, in plasma, four studies reported decreases in DHA, EPA, and AA levels, while one study indicated an increase in AA levels. Whole-blood analysis showed a decrease in DHA levels in one study, with no differences reported in EPA or AA levels. Erythrocyte membrane analysis demonstrated a decrease in DHA levels in three studies, an increase in AA levels in one study, and no differences in EPA levels across studies. These findings underscore the complex and diverse nature of fatty acid profiles in individuals with ADHD, with notable variations observed in different sample types (see Table 2).

### 3.1. Oxidative (and Nitrosative) Stress

Various processes occurring in the human body, such as mitochondrial activity; immune activation; inflammatory, ischemic, or infectious processes; and psychological stress, among others, lead to the formation of compounds with oxidative properties [115]. While initially, the focus was on oxygen-containing compounds, particularly “free radicals”, in recent decades, attention has shifted to a broader category of compounds with oxidative properties. These can be broadly categorized into two main groups: reactive oxygen species (ROS) and reactive nitrogen species (RNS) [116]. Additionally, reactive species can originate not only endogenously, but also from exogenous sources like pollutants, heavy metals, medications, diet, cigarette smoke, or alcohol [115].

*Effects of Oxidative Stress:* Extensively studied, these compounds have various harmful effects involving the destruction of major molecular classes such as proteins, lipids, and DNA [115]. To counteract these effects, the body possesses a range of antioxidant compounds. Oxidative stress occurs when the production of reactive species surpasses the defense capacity of the body’s antioxidant system. This phenomenon has been implicated in numerous psychiatric disorders, including schizophrenia, depression, bipolar affective disorder, obsessive–compulsive disorder, autistic spectrum disorder, and ADHD [52,53,117]. The brain seems predisposed to oxidative stress due to factors such as its lipid-rich content, high energy requirements, and low concentrations of antioxidants like glutathione [55,59]. Oxidative stress has been demonstrated to affect the structure and function of various substrates, including neuronal receptors, leading to subsequent alterations in the functioning of key neurotransmitter systems, proteins, and lipids. Overall, this contributes to a disruption in the nervous system’s operation [55]. However, data suggest that the impact of oxidative stress on the brain is not uniform, with large neurons in specific brain regions being more prone to oxidative lesions due to a phenomenon termed “selective neuronal vulnerability”. This may explain the association between oxidative stress and certain psychopathological manifestations [118].

*Measurement and Studies:* As a result of the action of reactive species on various substrates, degradation compounds are produced. Thus, measuring peripheral levels of these compounds reflects specific oxidative processes. Numerous studies have been conducted to measure the levels of reactive species, antioxidants, and degradation compounds resulting from oxidative stress associated with various conditions, including ADHD.

**Malondialdehyde (MDA).** Malondialdehyde (MDA) is the final product of oxidative reactions involving polyunsaturated fatty acids (PUFA) within lipid peroxidation processes [119]. Essential fatty acids, discussed in detail in the following section, play a crucial structural role in neurons, constituting cellular membranes and influencing their biological properties [64]. Two studies have demonstrated significantly elevated levels of MDA in children and adolescents with ADHD, suggesting a potential link to oxidative stress affecting neuronal membranes [53,55]. Moreover, authors of a study suggested that dopaminergic neurons could be particularly susceptible to such lesions, given dopamine’s predisposition to autooxidation in the absence of efficient antioxidant action. Supporting their hypothesis, the level of glutathione peroxidase, a crucial enzymatic antioxidant, was significantly reduced in the studied sample [53]. It is noteworthy that another study identified high MDA concentrations in children with ADHD, but this difference lost statistical significance after corrections were applied [62]. Two included studies did not find significant differences in MDA levels [52,58]. However, Avcil et al. noted in their study that measuring absolute values of various oxidative stress markers is often insufficient, suggesting the utility of calculating ratios between them. In the same study, a significant decrease in the MDA/melatonin ratio was identified, especially in patients with a family history of ADHD. The authors explained this difference by cumulative genetic load, suggesting that both oxidative and nitrosative metabolism are genetically influenced [52]. Interestingly, another study involving participants with learning disorders (LDs), some of whom had ADHD, identified a significant increase in MDA when comparing LD children with healthy ones. However, no significant differences were found between the group with comorbid LDs and ADHD and those with pure LD [93]. Additionally, two studies reported a significant decrease in MDA levels. One of them suggested that the high values of oxidative stress markers identified by other authors might be associated more with the older age of participants and exposure to various risk factors than with ADHD itself [60,75].

**8-Hydroxy-2′-deoxyguanosine (8-OHdG)** is another oxidative degradation marker that has been studied in ADHD. It results from the oxidation of guanosine within the DNA structure, but its values were found to be lower in only one study [60], while in two other studies. it did not differ significantly within the studied population [61,62].

**Thiols** represent a class of sulfur-containing compounds of particular significance due to their coordination role within the antioxidant system. They undergo oxidation by reactive species, leading to the formation of disulfide bonds, which are subsequently reduced by antioxidants. The concept of thiol–disulfide homeostasis is thus described, extending its involvement beyond antioxidant defense to include essential processes such as organism detoxification, apoptosis, cellular signal transduction, as well as the regulation of various enzymes and transcription factors [51]. A study involving 50 participants aged 6 to 16 with ADHD identified significantly diminished levels of native thiols, total thiols, and disulfides, along with a negative correlation of these thiols with inattention and hyperactivity scores. Additionally, a negative correlation of total thiols with the ADHD index on the Conners Parent Rating Scale was observed. From these findings, the authors suggested that although thiol–disulfide homeostasis is altered in ADHD, oxidative stress appears to be more pertinent for assessing ADHD severity than its subtyping [59]. Another study, including 52 children with ADHD, identified a thiol deficit that persisted significantly even after the administration of methylphenidate. The authors proposed that children’s antioxidant systems exhibit inefficiency under conditions of oxidative stress, failing to increase to a compensatory degree, as observed in adults [56]. Conversely, a study conducted by Avcil et al. in 2017 reported a substantial increase in both total and native thiols, interpreted as an attempt to counteract oxidative stress through a reduction in reactive species. Additionally, a decrease in the disulfide/native thiols ratio was identified, suggesting that disruption in this homeostasis could impact the dopaminergic system or lead to damage to certain brain structures [51]. Given the closely aligned demographic characteristics of participants in both studies and the mentioned considerations, we posit that disparities in the severity of participants’ conditions may contribute to this apparent contradiction. It is conceivable that the latter study incorporated milder forms of ADHD; however, this conjecture remains unverifiable due to the absence of reported scores from participants’ psychometric tests. Another study failed to identify noteworthy changes in thiols [60]. Furthermore, a study involving a cohort of 32 children with ADHD revealed diminished concentrations of glutathione, a low-molecular-weight thiol. The authors of this study contended that the children had not been extensively exposed to oxidative stressors. Nevertheless, nearly all patients included in the study were undergoing treatment with methylphenidate, which is acknowledged for its protective effects against oxidative stress [51,58].

The metabolism of nitric oxide has been subject to investigation in a series of studies. **Nitric oxide (NO)**, synthesized from the essential amino acid L-arginine under the action of nitric oxide synthase (NOS), is both a reactive oxygen and nitrogen species and a neurotransmitter in the nervous system, playing numerous roles, such as dopamine and norepinephrine release, modulation of memory, learning, and wakefulness [52]. NO is a highly labile molecule prone to decomposition into two stable oxidized final products: nitrites (NO_2_^−^) and nitrates (NO_3_^−^), whose plasma levels were reported as significantly elevated in children and adolescents with ADHD in two studies [53,57]. The authors of one study proposed several hypotheses to attempt to justify these differences. On one hand, there might be extrarenal nitric oxide synthesis in individuals with ADHD, potentially exacerbated by methylphenidate. On the other hand, changes in levels of asymmetric dimethylarginine (ADMA), an endogenous inhibitor of NOS whose concentrations were significantly reduced in one of the included studies, could be underlying these variations [57]. However, this latter hypothesis is contradicted by a recently published study demonstrating significantly increased levels of arginine and ADMA [54].

### 3.2. Lipid Metabolism

Polyunsaturated fatty acids (PUFA) play a pivotal role in the human organism. As emphasized earlier, they serve a structural role, constituting the membranes of all cells, including neurons, thereby modifying their function. Predominantly found in the brain are the omega-3 fatty acid docosahexaenoic acid (DHA, 22:6n-3) and the omega-6 fatty acid arachidonic acid (AA, 20:4n-6), accumulating in substantial quantities in the brain until the age of 5 and collectively representing 20% of the brain’s dry mass [65,72]. Additionally, the precursor to DHA, omega-3 fatty acid eicosapentaenoic acid (EPA, 20:5n-3), is also present [66]. In recent decades, an increasing number of neuroimaging studies have been conducted to correlate PUFA status with various brain modifications. It has been identified that elevated concentrations of DHA and EPA are associated with a larger volume of grey matter and cerebral blood flow, dependent on activity [68]. These modifications could be explained by the second primary role of fatty acids, alongside the structural role, namely, serving as precursors to eicosanoids—crucial mediators of biological processes involved in development.

It is fundamental to note that the effects of different types of fatty acids in the brain tend to be divergent. Arachidonic acid (AA) produces proinflammatory eicosanoids, whereas EPA gives rise to compounds predominantly with anti-inflammatory effects. For this reason, numerous studies have explored potential dietary influences on the balance between various types of PUFA, with evidence suggesting that the predominance of PUFA n-6 in all diets, especially in Western diets, may lead to an imbalance favoring the production of proinflammatory prostaglandins. Additionally, studies indicate that DHA influences intracellular signaling processes and neurotransmitter release by affecting the membrane fluidity of neurons [66].

*Involvement of Fatty Acids in the Pathophysiology of ADHD.* The implication of fatty acids in the pathophysiology of ADHD seems to be suggested by several cues. Firstly, it started with the high-frequency observation of specific signs and symptoms characteristic of PUFA deficiency among children with ADHD [36]. Subsequently, genomic studies revealed that PUFAs are not only phenotypically associated with ADHD, but also share some common genetic pathways [69]. Finally, neuroimaging studies demonstrated that the cortical effect of DHA appears to manifest predominantly in the frontal lobe, which is responsible for processes such as executive function, behavioral regulation, and emotions—all frequently implicated in the pathophysiology of ADHD [68]. Given these connections, several recommendations regarding fatty acid supplementation in children diagnosed with this condition have been formulated. However, a meta-analysis published in 2021 did not identify any benefits of such approaches in the treatment of ADHD [120].

It is important to highlight from the beginning that we have observed notable heterogeneity in the methodologies employed in studies conducted over time to investigate potential associations between polyunsaturated fatty acid (PUFA) levels and ADHD. Notably, variations exist in the targeted substrates; one study measured concentrations of free plasma fatty acids, while others focused on their levels in various lipid types—some exclusively in phospholipids, others in all lipid types—derived from either plasma or erythrocyte membranes, following pre-extraction using diverse techniques. The literature indicates that these concentrations exhibit distinct variations depending on the substrate, influenced by dietary factors and endogenous metabolic processes. Generally, plasma levels are considered to reflect the short-term lipid status, while membrane concentrations elucidate the effects of long-term accumulation [66,121]. Additionally, there are studies that have measured fatty acids in whole blood, with some authors deeming this approach more relevant than analyzing erythrocytes to reflect circulating PUFA status (both long-chain and short-chain) correlated with dietary intake [66]. Moreover, there is heightened heterogeneity in the reported laboratory parameters, with certain studies presenting only total values of omega-3 and omega-6 or even total PUFA, while others provided an extensive panel of all identified fatty acids. Furthermore, the methodologies of studies investigating fatty acids in ADHD also vary based on the degree of control over patients’ fat intake before testing. Considering all these aspects, our focus within this section is on results related to indicators found in most studies (as presented in Table 2), and, although not as extensively studied, they could offer new avenues for research.

Summarizing these results, several studies seem to indicate a deficit of DHA and AA among children and adolescents with ADHD, which could suggest a reduced synthesis of eicosanoids and, subsequently, neuroinflammatory processes. However, this interpretation is not unanimous, as some argue that these low levels are observed precisely due to an intense metabolism towards eicosanoids [64]. On the other hand, another potential cause suggested for the decrease in DHA levels was the high rate of fatty acid oxidation, highlighting a possible interplay with oxidative stress [65]. As can be seen from Table 2, not all studies have replicated these results, with one study identifying elevated levels of these fatty acids. However, the authors argue that behind these results, there could be population, dietary, genetic, or fatty acid metabolism characteristics [75].

An intriguing difference that could be relevant in the pathogenesis of ADHD is the significant decrease in nervonic acid in the phospholipids of the erythrocyte membrane, identified in the study led by Chen [64]. Building upon this discovery and considering that the levels of this compound in the erythrocyte membrane, which are known to be correlated with those in the brain, can provide information about the cerebral maturation of premature infants, the authors hypothesized that it could reflect the degree of cerebral maturity in patients with ADHD [64]. However, reports of this fatty acid have not been provided by other studies.

Several authors have attempted to correlate different modifications identified in the lipid profile of patients with specific phenotypes of ADHD or other variables. For instance, one study demonstrated that an elevated ratio between AA and DHA is associated with increased severity of behavioral symptoms, with a reduced relationship with cognitive changes [66]. Conversely, a neuroimaging study identified an inverse effect of EPA concentrations measured in whole blood on oxyhemoglobin levels in certain cortical regions during tasks evaluating selective attention [68]. Another study positively correlated the clinical status of female patients, but not males, with the ratio of omega-3 fatty acids to total plasma fatty acids. The same authors observed distinct lipid profiles between the two genders, suggesting possible sex-related alterations in PUFA metabolism, likely justified by genetically dictated changes in enzymatic activities [69]. Additionally, the overall severity of ADHD manifestations was negatively correlated with EPA and DHA levels in various plasma and erythrocyte membrane lipid substrates, with the note that only DHA in the form of plasma phosphatidylcholine was separately negatively correlated with both inattention and hyperactivity [71]. Another recent study also reported a negative correlation of DHA with inattention [72], but there was a study where the levels of DHA, EPA, and AA were positively correlated with inattention [74]. It is relevant to note that a study in which no statistically significant differences were identified after statistical corrections suggested that changes in long-chain PUFA concentrations could be associated with antisocial and psychopathic traits of children with ADHD, rather than manifestations of the disorder per se [67].

A relatively frequently debated topic has been the possible influence of different dietary patterns characteristic of patients with ADHD, but the results of several studies seem to challenge this idea [64,70,72,79]. A relevant study in this regard included 24 Japanese participants, starting from the premise that Japan has a high fish consumption, rich in fatty acids, and expecting to find no reduced levels of PUFA, as is often seen in other samples. Despite the authors’ expectations, their study highlighted significantly decreased plasma concentrations of DHA and EPA [79]. Another representative study led by Miklavcic et al. in 2023 identified an AA deficit despite significantly higher intake in 103 children with ADHD. The study’s authors suggested two potential causes, namely, polymorphisms in the fatty acid desaturase gene cluster (FADS) identified as risk factors for ADHD or increased conversion of arachidonic acids into various metabolites involved in neuroinflammatory and oxidative stress processes [72].

An alternative perspective is presented in a study that detected notable alterations in the levels of sphingolipids within a cohort of 28 ADHD patients aged 5 to 18 years. Abundant in nervous tissue, sphingolipids play a role in influencing various cellular processes, including cell proliferation, differentiation, and apoptosis, as well as membrane permeability, nerve impulse transmission, and neurotransmitter release. Drawing from these findings, the authors propose the potential use of sphingomyelin C24:1 and deoxy ceramide C24:1 as markers for identifying ADHD endophenotypes [70].

### 3.3. Amino Acid Metabolism

Amino acids are organic substances containing both amino and carboxyl groups. While over 300 amino acids have been identified, only 20 of them serve as structural units for protein synthesis. All these 20 amino acids, along with their metabolites, are essential for the proper functioning of the body, as disruptions in a single amino acid can potentially impact a variety of crucial processes. Furthermore, amino acids not involved in protein formation (non-protein amino acids) also play fundamental roles in cellular metabolism [122].

**Homocysteine** is a non-protein, sulfur-containing amino acid with low molecular weight, resulting from the metabolism of methionine [51,80]. The significance of these two amino acids is suggested primarily by their common involvement in the main one-carbon metabolism pathways, which encompass methylation, transsulfuration, and folate metabolism. The proper functioning of these pathways is crucial from early life, contributing to fetal development [123]. The interest in studying these elements in psychiatric disorders is justified by ample literature support. Firstly, elevated homocysteine levels exhibit neurotoxic effects through the activation of NMDA receptors in the glutamatergic system and increased oxidative stress, posing a risk factor for various conditions, including autism spectrum disorder, bipolar disorder, and ADHD [80,124,125]. Additionally, the metabolic products of homocysteine can induce neuronal damage, affecting protein synthesis and neurotransmitter function [89].

Surprisingly, one of the studies that investigated plasma homocysteine levels in a sample of 30 participants with ADHD, aged between 6 and 15 years, reported a significant decrease compared to the healthy control group. The authors suggested that this apparent homocysteine deficiency could contribute to oxidative stress in ADHD by reducing cysteine formation through the transsulfuration pathway, subsequently leading to decreased synthesis of glutathione, one of the body’s primary antioxidants [80]. These findings imply a limited response to oxidative stress in children with ADHD, aligning with discussions in the oxidative stress section [56]. However, this result was not replicated by another study, which identified significantly elevated plasma homocysteine levels positively correlated with symptoms of hyperactivity and impulsivity. These increased levels were attributed to low vitamin B12 levels among the participants. Furthermore, the authors highlighted the involvement of vitamin B12 in the dopamine-stimulated phospholipid methylation process, conducted by certain dopamine receptors frequently associated with ADHD, playing a crucial role in attention modulation [89] Another study reported non-significant differences in homocysteine levels but failed to provide details on the statistical tests used or the laboratory methods applied [86].

**Aromatic amino acids** (tryptophan, tyrosine, phenylalanine, and histidine) stand out due to their crucial role as prerequisites in the synthesis of key neurotransmitters implicated in ADHD—serotonin (synthesized from tryptophan), dopamine (synthesized from tyrosine, which can be obtained through the hydroxylation of phenylalanine), norepinephrine, and epinephrine (sequentially synthesized from dopamine) [126]. They also play a role in other neurotransmitters with potential relevance to ADHD, such as histamine (synthesized from histidine), which modulates various processes, including motivation, sleep, and wakefulness [126]. Significant decreases in the plasma concentrations of these amino acids were identified in a study from 1990 involving 28 children with ADHD, suggesting a potential deficit in the transport and/or absorption of amino acids that compete to cross the blood–brain barrier. However, the same study failed to identify significant changes in the urinary concentrations of phenylalanine and tyrosine, the only amino acids for which urinary values were reported [81]. Similar results, demonstrating reduced plasma concentrations of phenylalanine and tyrosine without corresponding changes in their urinary excretion, were reported in another study [98]. Another more recent study, which included a larger sample of participants (n = 83) without excluding those with various comorbidities or undergoing medication, did not identify differences in plasma or urinary levels of aromatic amino acids. The authors theorize that the neurotransmitter alterations observed in ADHD may not stem from deficits in precursor aromatic amino acids, but rather from an altered transport across the blood–brain barrier or abnormal reuptake. An alternative explanation provided by the authors is that aromatic amino acid levels must drop below a certain threshold to impact behavior [82]. Insignificant differences also emerge from the analysis of results published by Skalny et al. in 2021, although the authors of this study presented changes in histidine as significant despite a *p*-value > 0.05. However, the same study identified an excess of **hydroxyproline**, interpreted as a marker of associated connective tissue pathology, based on observations from other studies indicating that patients with ADHD more frequently exhibit joint hypermobility. Additionally, changes in glutamine (decreased) and glutamate (increased) are identified, along with a significant decrease in their ratio, highlighting the alteration of the glutamatergic system often implicated in ADHD [87].

A concept similar to the one proposed by Bergwerff et al., suggesting that there is a threshold below which amino acids must drop to impact the nervous system’s functioning, is also evident in another study. This study not only identified a significant increase in L-cysteine levels in children with ADHD, but also found that the values of this amino acid correlated negatively with neuroimaging changes specifically in these children, not in healthy ones. Specifically, the study revealed that, in functional magnetic resonance imaging, children with ADHD exhibited a greater disconnect between the default-mode network and its components on one hand and regions influenced by the salience network on the other as homocysteine levels increased. This connectivity imbalance between two essential brain systems could underlie the marked distractibility observed in ADHD. It is suggested that L-cysteine levels may increase as a result of the immaturity of these circuits, attempting to compensate for it, given the neuroprotective and signaling effects of this amino acid [88]. Alterations in amino acid and/or neurotransmitter metabolism were also suggested by a study involving 100 children with ADHD aged between 2 and 12 years. The study identified a significant increase in plasma levels of lactate and ammonia, both potential degradation products of the mentioned substrates. However, the study presented two major limitations that could influence the results, namely, comparing marker results with laboratory reference values rather than those recorded in a control group and a very high rate of consanguinity and minimal brain damage [83]. Additionally, a multi-omics integrative study published in 2024 identified a correlation pattern in which only amines (mostly essential amino acids) were negatively correlated with certain CpG sites whose methylation may be implicated in ADHD pathogenesis. The same study identified a correlation pattern that included, among other things, the polygenic score transmitted for ADHD and 10 amino acids, pinpointing a gene not previously linked to ADHD but specifically associated with smoking, aging, and type II diabetes [84]. Finally, there is a study asserting that in patients with ADHD, whose number is not specified, a urinary tetrapeptide was identified that is absent in healthy subjects (but was detected in 2 out of 10 autistic children), without interpreting these results in terms of potential explanatory mechanisms [85].

### 3.4. Kynurenine Pathway

As evident, amino acid metabolism is intricately connected with numerous other processes, such as the previously discussed oxidative stress, and neurotransmitter metabolism, which, although mentioned several times, will be systematically discussed later. Additionally, another amino acid, tryptophan, serves as the starting point for the kynurenine pathway, undergoing a 90% conversion to L-kynurenine [109]. Following the reactions in this pathway, compounds capable of influencing central nervous system function in various ways are produced, depending on which of the three possible routes is taken by L-kynurenine. The key distinguishing compounds include kynurenic acid (with neuroprotective and anticonvulsant effects); 3-hydroxykynurenine; through its metabolism, quinolinic acid (an excitotoxic product which serves as a direct precursor of nicotinamide); and anthranilic acid [91]. Significant influences on the kynurenine system come from psychological stress and chronic inflammation, which, through elevated cortisol levels, activate enzymes involved in the system. The consequences of this activation include heightened oxidative stress, increased cytokine production, neuroinflammation, reduced neuroplasticity, and disruption of the balance between neurotransmitters, affecting the activity of dopamine, acetylcholine, and the glutamatergic system, particularly by impacting NMDA receptors [93,94]. Furthermore, oxidative stress seems to lead to a preferential metabolism of tryptophan through the kynurenine pathway, establishing a vicious cycle that intertwines these two major processes [93].

Numerous studies have been conducted to investigate the involvement of this metabolic pathway in ADHD. An intriguing initial study was led by Dolina, aiming to test hypotheses suggesting that ADHD, much like epilepsy, could be considered an inborn error of vitamin B6 (pyridoxine) metabolism. This hypothesis stems from the fact that pyridoxine is a coenzyme that controls numerous steps in amino acid metabolism and every step in the kynurenine pathway. The results of this study indicated, among other findings, significant increases in plasma levels of tryptophan, kynurenine, 3-hydroxykynurenine, and kynurenic acid in untreated children with ADHD. In contrast, medicated participants did not show significant differences in the latter two indicators compared to the control group. According to the authors, this tryptophan degradation profile was nearly identical to that in epilepsy, suggesting a common biochemical profile of the two frequently comorbid pathologies, characterized by a global deficit in pyridoxine utilization leading to alterations in the kynurenine pathway, amino acid metabolism, and neurotransmitters [90]. Elevated levels of tryptophan and kynurenine were also reported in a study involving 102 non-medicated children with ADHD, along with a deficit in kynurenic and anthranilic acids. Additionally, the deficit of anthranilic acid exhibited a particularly diagnostic value (AUC = 0.88). The study’s authors conducted a post hoc analysis where ADHD participants were divided into two groups based on the kynurenine/tryptophan ratio, significantly elevated across the entire sample, demonstrating that the anthranilic acid deficit can be observed even when L-kynurenine is generated in adequate amounts, suggesting a deficient activity of enzymes involved in this pathway [91]. Another study identified an excess of free plasma tryptophan, correlating with the severity of ADHD [92].

Not all studies, however, have identified significant changes in metabolites resulting from the kynurenine pathway [93,94]. One of these studies included, in addition to a group of 54 healthy children, 100 participants with learning disorders, of which 31 had comorbid ADHD. Although the overall LD group showed significantly elevated levels of kynurenine compared to healthy children, this difference was attributed to the psychological stress these children undergo due to their pathology. However, when comparing the two groups of children with LD, no significant differences were observed [93]. The second study presented the limitation of including the siblings of ADHD patients as the control group, thus carrying the risk of confounding variables such as common genetic and environmental factors. Nevertheless, an interesting result of this study is that depressive symptoms were the main factor influencing the direction and magnitude of changes in kynurenic markers [94].

A surprising discovery was made in the study led by Oades et al., which identified significantly increased levels of neurotoxic 3-hydroxykynurenine in healthy children. One hypothesis proposed to justify this result suggests that toxic metabolites from the kynurenine pathway might actually be a normal feature in late childhood, indicating neural maturation processes, while lower levels could reflect a delay in nervous system development [95]. This hypothesis is supported by high levels of oxidative stress and immune activation during brain development, inducing the activity of kynurenine pathway enzymes [94]. A follow-up of this study revealed a lack of significant correlations between tryptophan metabolites and ADHD symptoms per se, although high tryptophan concentrations predicted a higher frequency of omission errors, and kynurenine concentrations predicted shorter reaction times in Continuous Performance Task measures [96]. Another study including a healthy control group and three groups of participants with ADHD—one without comorbidities and the others with oppositional defiant disorder and conduct disorder, respectively—also identified lower serum concentrations of 3-hydroxykynurenine than in healthy children. However, this was observed only in the 46 participants with non-comorbid ADHD, as the 33 children and adolescents with ADHD comorbid with conduct disorder did not show this decrease. Additionally, serum kynurenine was increased only in the 46 participants with non-comorbid ADHD. Thus, the authors suggest that metabolites of the kynurenine pathway seem to reflect unique biological characteristics of children with ADHD unaccompanied by comorbidities; otherwise, they lose their utility. Somewhat surprisingly, although not significantly altered, kynurenic acid values were positively correlated with the anxiety scores reported by the children [97].

### 3.5. Neurotransmitter Metabolism

In the pathophysiology of ADHD, dysfunctions in the major neurotransmitter systems have been implicated. The most extensively studied theories are those related to dopamine and norepinephrine, supported by a wealth of research data suggesting their involvement. These include the abundance of specific subtypes of dopaminergic receptors in reward, memory, learning, and motor activity circuits; an increase in presynaptic dopamine reuptake in individuals with ADHD due to the high density of the dopamine transporter; the role of norepinephrine in modulating working memory; and the favorable effects of stimulant medication, such as methylphenidate, which inhibits monoamine reuptake [127]. Moreover, given its multiple influences on aspects highly relevant to ADHD, such as attention, impulsivity, and aggression, the serotonergic system has also been proposed as a potential mechanism in the pathogenesis of the disorder [100]. Some authors have even suggested the potential involvement of other systems, such as the glutamatergic and GABAergic systems [127]. In light of these considerations, it has been deemed that measuring peripheral levels of metabolites of these neurotransmitters could be an easily assessable method for evaluating characteristic imbalances in children and adolescents with ADHD. Due to their stability, sensitivity, and non-invasive nature, urinary assessments have become the primary approach in this regard [128]. It is important to note, however, that urinary concentrations of various neurotransmitters and their metabolites can originate from multiple sources, both neuronal and extra-neuronal (e.g., dopamine synthesis in renal tubules) [101]. Thus, it is estimated that up to 50% of urinary 3-methoxy-4-hydroxyphenylglycol (MHPG), the principal metabolite of norepinephrine, originates from cerebral metabolism of this neurotransmitter [102]. Reports on urinary levels of this compound in children with ADHD vary, with some studies not identifying significant differences [99,105] and others attesting to a modified urinary excretion, either increased [102] or decreased [107]. It is noteworthy that MHPG is derived from another compound, 3,4-dihydroxyphenylglycol (DOPEG), obtained through the oxidative deamination of norepinephrine, and its urinary levels have also been significantly reduced in children with ADHD. The authors of this study justified the results by a possible alteration of either norepinephrine reuptake or its oxidative deamination. However, it is also possible that anomalies in the excretion of these noradrenergic metabolites indicate dysfunctions of the sympathetic nervous system or renal hemodynamic factors without being directly linked to neuronal activity [101].

In comparison to MHPG, metanephrine and normetanephrine are metabolites that reflect the extracerebral status of adrenaline and norepinephrine. Two studies on this subject reported nonsignificant differences [99,102], while a more recent study identified an increased urinary excretion of normetanephrine in children with ADHD and those who had experienced traumatic brain injuries (TBI) compared to a group of healthy children. The rationale behind including these two patient groups is based on data confirming a similar behavioral profile in ADHD and TBI, with neuroimaging studies confirming the preferential involvement of frontal brain lobes in such traumas, involving changes in the prefrontal and striatal cortex, as well as a similar response to methylphenidate treatment. However, differences seem to lie in the fact that, although both patient categories exhibit tonic hyperactivity of the noradrenergic system, only those with TBI also show an exacerbation of adrenergic activity [103].

Interestingly, our search identified only two studies that measured the levels of homovanillic acid, the main metabolite of dopamine. One of them showed a decrease in its urinary excretion in 28 participants with ADHD [107], while the other one did not find any significant differences [105]. However, the utility of these results is limited, considering that only 33% of urinary homovanillic acid is derived from dopamine metabolism in the central nervous system [107].

Serotonin, produced within one of the metabolic pathways of tryptophan, later transforms into either melatonin or 5-hydroxyindoleacetic acid (5-HIAA). It is noteworthy that approximately 95% of the total serotonin quantity is synthesized in the gastrointestinal tract, with only 5% in the central nervous system [109]. A recent study identified a reduced concentration of 5-HIAA in the plasma of 35 children with ADHD compared to a group of 26 healthy participants, although the actual levels of plasma serotonin did not differ. These results were attributed to the population characteristics of the Indo-Caucasian sample, incriminating their dietary habits and genetic factors that favor, on the one hand, rapid serotonin reuptake and, on the other hand, inadequate serotonin metabolism characterized by low turnover [100]. Regarding urinary concentrations of this metabolite in children with ADHD, literature reports are heterogeneous, with evidence showing both lower [104] and higher [105] levels, as well as a study that did not identify significant differences [95].

In addition to studies focusing on the metabolites of the main neurotransmitters implicated in ADHD, two works have examined the levels of biogenic amines known to modulate neurotransmission. One such study targeted the measurement of tetrahydroisoquinoline derivatives (TIQ). Two of these compounds, salsolinol and norsalsolinol, are synthesized in the human brain from dopamine and influence the status of catecholamine receptors, as well as the activity of enzymes involved in the biosynthesis of these neurotransmitters. Their maximum concentration is reached in the basal ganglia, an area incriminated in the etiology of ADHD due to its involvement in multiple functions, including motor control, a fact supported by animal studies where exogenous TIQ administration produced changes in motor activity [106]. Roessner et al. identified increases in urinary concentrations for four free TIQ, a relevant aspect given that urine levels seem to be correlated, according to some authors, with those in cerebrospinal fluid. Furthermore, urinary concentrations of another TIQ, N-methyl-salsolinol, identified ADHD with a sensitivity of 92.5% and a specificity of 94.4%. The results obtained seem to indicate a hyperdopaminergic status [106]. Although such a hypothesis contradicts the bulk of the literature on this subject, there are other works attempting to nuance the dopaminergic theory of ADHD, with one suggesting that differences dictated by the involved brain region may exist (e.g., dopaminergic excess in the striatum and accumbens vs. dopaminergic deficit in the limbic system) [129]. Another study supports the idea that in ADHD, dopamine may be in excess in relation to noradrenergic metabolism, but at the same time deficient when compared to serotonin metabolism [130]. Considering all the presented aspects, the results seem to indicate that neurotransmitter disruptions in children and adolescents with ADHD involve qualitative disturbances in the action of different neurotransmitters rather than simple changes in their absolute values. This leads to a deficient orchestration of various neurochemical systems. Finally, another study measured the urinary levels of phenylacetic acid, the main metabolite of phenylethylamine (a minor neurotransmitter), without, however, identifying significant variations compared to the control group [98].

### 3.6. Other Metabolic Processes

#### 3.6.1. Melatonin Metabolism

As mentioned earlier, melatonin has serotonin as its precursor. Given the high prevalence of sleep disorders and daytime sleepiness among children and adolescents with ADHD, Büber et al. investigated the levels of the main urinary metabolite of melatonin, 6-hydroxymelatonin sulfate (6-OH-MS), demonstrating significantly increased excretion regardless of the time of day. This suggests a high catabolism of melatonin. Furthermore, the authors argue that ADHD might involve an exaggerated production of melatonin at both the pineal (attributing it to a GABA deficit in this case) and extra-pineal levels. One hypothesis suggests that disruptions in melatonin synthesis could be caused by frequent sleep interruptions due to paroxysmal electroencephalographic anomalies. These anomalies are more common in children with ADHD than in healthy ones, reinforcing possible connections between this disorder and epilepsy [108]. Melatonin excess was reported in a study involving 103 children with ADHD [52]. In contrast, another study with 136 participants diagnosed with ADHD did not identify significant differences compared to the control group. It only found a significant increase in those with predominantly hyperactive ADHD or ADHD associated with conduct disorder, compared to those diagnosed with predominantly inattentive ADHD. The same study also indicated a high excretion of 6-sulfatoxymelatonin, another melatonin derivative, but this difference lost its statistical significance after age correction [110]. It is worth mentioning that, in addition to its essential role in regulating the circadian rhythm, melatonin, along with its metabolites, serves as an indirect antioxidant through the scavenging effect on reactive oxygen and nitrogen species. Melatonin demonstrates increased resistance against these species. Moreover, due to its rapid inducibility and amphiphilic properties, melatonin provides better protection to the brain than other antioxidants [52]. In addition to its interactions with neurotransmitter metabolism and oxidative stress, melatonin is also associated with the kynurenine pathway, influencing its activity [93].

#### 3.6.2. Indole Tryptophan Metabolism

Tryptophan that does not follow one of the three previously described pathways (i.e., incorporation into proteins, the kynurenine pathway, serotonin synthesis) is broken down by the intestinal microbiota, outlining the fourth pathway of tryptophan metabolism with the formation of indolamines. These metabolites serve various functions, such as maintaining bidirectional communication between the brain and the intestine, neuroprotection, intestinal barrier protection, and antioxidant function [109,131]. A study published in 2020, involving a sample of 107 children with ADHD aged between 5 and 15, investigated the plasma and urine levels of indolamines (indoleacetic acid, indolpropionic acid) and tryptamine, a byproduct of tryptophan degradation. The study did not identify significant differences in these levels [109].

#### 3.6.3. Agmatine

Agmatine, a polyamine endogenously produced from L-arginine, plays a critical role in cognitive functions, emotional processing, and the regulation of oxidative stress. It reduces nitric oxide levels by inhibiting its cerebral synthesis. This amine has been studied in various psychiatric disorders, including autism spectrum disorder, and preclinical studies attest to the beneficial effects of agmatine on functions such as learning and working memory [111]. A study involving 35 children with ADHD aged between 6 and 14 identified significantly increased plasma levels of agmatine and its precursor, L-arginine, suggesting that this could be a compensatory mechanism aimed at improving executive function deficits in ADHD by inhibiting nitric oxide and glutamine synthesis [111].

### 3.7. Metabolomic Untargeted Studies

Hippuric acid is a bioactive compound resulting either from the hepatic metabolism of benzoic acid, typically produced by the action of intestinal microbiota on polyphenol-rich foods, or from the intestinal microbial metabolism of phenylalanine [132]. A recent untargeted urinary and fecal metabolomics study identified significantly higher levels of hippurate in the urine of boys with ADHD, along with a negative correlation of this concentration with IQ, but this was not found among girls. Additionally, it was correlated with other microbial metabolism products. However, this study had some limitations, such as including participants in the control group up to 22 years old and including twin pairs where one had ADHD and the other did not, which could have influenced the results due to shared genetic and environmental factors [112]. It should be noted that another untargeted metabolomics study had previously identified significant increases in plasma 4-aminohipuric acid, a derivative of hippuric acid, and its correlation with symptoms [114].

Another untargeted metabolomics study is the one published by Tian et al. in 2022. This study included 76 children with ADHD, with or without tics, and 363 healthy participants, dividing them into five age categories to investigate variations in metabolites throughout ontogenesis. Initially, metabolic profiles were analyzed based on gender, identifying the gender-dependent nature of several metabolic processes presented in this review. Arachidonic acid metabolism appeared to be gender-dependent in 4–6-year-old children, glutamate and fatty acids in 7–10-year-olds, and catecholamine synthesis in 15–18-year-olds. Furthermore, age-dependent differences were identified among participants of the same gender. As children age, boys undergo changes in catecholamine, fatty acid, and amino acid metabolism, while girls undergo alterations in other processes, such as tryptophan metabolism. Additionally, boys exhibited higher concentrations of carnitine and its metabolites, suggesting greater use of fats in the energy metabolism of males, given the involvement of this compound in fatty acid oxidation and branched-chain amino acid metabolism. Moreover, 34 metabolites were identified that distinguished between children with ADHD without comorbid tics and healthy participants, most of which were related to amino acid metabolism (especially tyrosine and tryptophan) and fatty acids. Lastly, the authors identified a panel of three metabolites (FAPy-adenine, 3-methylazelaic acid, and phenylacetylglutamine) that could predict ADHD with very high accuracy (ROC-AUC = 0.916) [113].

Finally, a recent untargeted plasma metabolomics study identified nine metabolites that differentiated the 58 participants with ADHD from the 38 healthy individuals, including four compounds not previously implicated. One such compound, with significantly elevated plasma concentrations, was 5-hydroxylysine, a hydroxylated derivative of lysine identified in various types of collagens, with inborn metabolic errors associated with changes in brain structure. The other three compounds were L-cysteine, previously discussed in the Amino Acids section; gentisic acid; and tryptophyl-phenylalanine. Significant correlations with ADHD symptomatology were observed for all these compounds, as well as for o-phosphoethanolamine, which serves as both a precursor for phospholipid synthesis and a product of their catabolism, with increased levels during periods of neuritic proliferation. This compound exhibits a high structural similarity to GABA and appears to influence cholesterol metabolism and to be involved in the development of ADHD-like behaviors [114].

## 4. Conclusions

A close look at the metabolomic markers identified so far in children and adolescents with ADHD reveals a multifaceted landscape in which five main metabolic pathways (i.e., oxidative and nitrosative stress, fatty acid metabolism, amino acid metabolism, neurotransmitter metabolism, and the kynurenine pathway) intertwine in a complex and fascinating manner, although at first glance, they may appear totally distinct. Thus, we have shown how very diverse processes can converge to potentiate oxidative stress, such as the oxidation of fatty acids in the brain; the elevation of levels of amino acids such as homocysteine, with subsequent activation of neurotransmitter receptors; or falling into a vicious cycle of mutual potentiation between oxidative stress and preferential tryptophan metabolism via the kynurenine pathway. Moreover, we have tried to highlight markers that, although related to the five main metabolic pathways, cannot truly be classified into any of them. In this regard, we have presented, among others, how changes in melatonin metabolism could not only explain the sleep changes frequently seen in children with ADHD, but also constitute an important link to the oxidative balance and the kynurenine pathway. Last but not least, we also mentioned how the metabolic compounds of the gut microbiota might be involved in maintaining the body’s oxidative balance. With all this in mind, it seems that in trying to unravel what is going on in ADHD, much of the researchers’ attention should be focused on oxidative and nitrosative stress processes.

The importance of studying metabolomic markers in ADHD, however, goes far beyond pure interest in a better understanding of the pathophysiology. Unraveling the involved mechanisms constitutes only a precursor to other goals. Although equivocal, the results in the current literature suggest potential diagnostic markers, not only for ADHD treated globally (with tetrahydroisoquinoline derivatives being able to depict ADHD with an impressive sensitivity and specificity of over 90%), but also for its different facets. Thus, markers such as homocysteine appear to be associated with hyperactivity/impulsivity and disulfides with the combined subtype of ADHD, while kynurenine and total thiols have been correlated with inattention. The predictive ability of different biomarkers goes even further on transdiagnostic manifestations (e.g., callous-unemotional traits could be predicted by low levels of EPA) or on neurofunctional changes that could account for ADHD-associated changes (e.g., a deficient connectivity between regions of the default mode network and the salience networks has been negatively correlated with L-cysteine levels). Furthermore, some markers might indicate the association of comorbidities that, although commonly found in these patients, might otherwise go unnoticed (e.g., depression, which seems to be a factor that strongly influences kynurenic markers).

In addition to the prospect of improving diagnostic algorithms in a pathology that continues to be problematic in clinical practice, identification of these disturbances could facilitate targeted therapeutic interventions tailored to the phenotype, whether those involve the simple supplementation of relatively common compounds such as fatty acids or amino acids or more complex treatments that address brain-level biochemical alterations, such as those between neurotransmitters.

Far from considering that this systematic review elucidates these issues, we nevertheless believe that it could provide a starting point for further research. Metabolomics is still a young science, as related technologies and statistical tools are constantly evolving. Hope comes from studies conducted in recent years using untargeted metabolomics or multi-omics approaches, which open new horizons in research in this field and offer the prospect of a more accurate mapping of the relationships within this complex set, taking into account variations dictated by a multitude of factors such as age, gender, comorbidities, clinical phenotype, genotype, and environmental factors. Thanks to such approaches, some markers, most of them amino acids, have been identified that could be directly associated with the polygenic ADHD risk score. In addition, the potential involvement of genetic factors that had previously only been implicated in other disorders was discovered. Beyond these results, there are still many issues that need to be revealed in order to make more sense of the results gathered so far in the literature. Although several studies suggest the probable involvement of certain genetic factors in the imbalances of the five main metabolic pathways mentioned above, these remain, at present, mere assumptions. Therefore, we believe that the top priority in this area should be the conduct of collaborative, multi-center studies with larger sample sizes, so that they could provide more comprehensive insights into the metabolic intricacies of ADHD.

In addition to that, essential directions for future research should also include conducting longitudinal studies to analyze how various metabolomic markers vary throughout ontogenesis, conducting integrative multi-omics research considering multiple potentially implicated factors, and utilizing machine-based learning techniques to identify metabolomic clusters among children and adolescents with ADHD. Regardless of the study type, future research should prioritize standardized diagnostic criteria, ensuring that participants meet rigorous ADHD diagnostic standards. Additionally, efforts should be directed towards minimizing demographic heterogeneity to enhance the generalizability of the findings. Standardization of analytical techniques and methodologies will be pivotal for facilitating comparisons across studies and establishing a more cohesive understanding of metabolic dysregulation in ADHD. In this regard, we find it beneficial to develop a protocol guiding the consistent conduct of future studies on this specific subject, and in psychopathology in general.

**Limitations.** The study has several limitations that warrant consideration. First and foremost, the included studies exhibit an extremely high degree of heterogeneity in various aspects. On one hand, the ages of children with ADHD varied considerably, and some studies did not present the actual age range, but only the mean age and standard deviation. The age of participants in the control group was even more variable, with some studies comparing values in children with ADHD to those obtained from samples that included adults. Furthermore, the control groups in different studies were highly diverse, including healthy participants selected from the general population in some cases, and in others, relatives of children with ADHD or patients with ADHD comorbid with other conditions. Moreover, several studies did not have a proper control group, but instead used laboratory reference values for comparisons. Moreover, the assessment of the target group varied among studies. While the majority included participants diagnosed with ADHD by specialists, some studies only subjected them to psychometric assessments. Another aspect that varied considerably was the laboratory techniques used, with multiple methods employed, some of which (e.g., gas chromatography, thin-layer chromatography) gradually lost their utility, especially in the context of large-scale untargeted metabolomic testing. This technical heterogeneity complicates the synthesis of findings and limits the ability to compare results consistently. Furthermore, the details provided in some studies regarding the applied methodology were insufficient for an accurate assessment of its quality. Finally, the statistical procedures used differed considerably between studies, emphasizing that not all studies controlled for variables that could have influenced the results.

Regarding our review itself, mainly due to space considerations, but also due to the vastness of the subject, it was almost inevitable to omit markers that, although possibly relevant, were not reported frequently enough to stand out, particularly in lipid profile studies, where numerous studies presented dozens of variables. Moreover, it is possible that some studies were omitted from our search string or were lost in the initial result screening process. Additionally, although constituting a highly interesting and promising research direction with the potential to pave the way for prevention and/or early intervention, studies focusing on prenatal markers correlated with ADHD were not included in our work.

## Figures and Tables

**Table 1:** Characteristics of the studies included in the systematic review.

Study	Sample Characteristics	Results	Significant Correlations
**Oxidative stress**			
**Avcil, 2017 [51]**	**Group 1 (ADHD):** n = 90; age 6–14 (10.26 ± 2.35 years); M:F = 72:18; without medication; without comorbidities. **Group 2 (HC):** n = 65; age range not specified (10.86 ± 1.89 years); M:F = 52:13; not mentioned whether they were screened for psychopathology.	*Serum* - ↑ total thiols, native thiols - ↓ disulfide/native thiol ratio - no statistically significant difference for disulfide, native thiol/total thiol ratio, or disulfide/total thiol ratio	- ↑ disulfide in males - ↑ disulfide, disulfide/native thiol ratios in ADHD combined type - no significant correlations with parameters of ADHD severity
**Avcil, 2021 [52]**	**Group 1 (ADHD):** n = 105; age range not specified (9.45 ± 1.32 years); M:F = 78:25; without medication; without comorbidities. **Group 2 (HC):** n = 73; age range not specified (9.78 ± 1.02 years); M:F = 55:18; screened for psychopathology.	*Serum* - no statistically significant difference for MDA - ↓ MDA/melatonin ratio (especially in those with a family history of ADHD)	- no significant correlations with parameters of ADHD severity
**Ceylan, 2010 [53]**	**Group 1 (ADHD):** n = 35; age 7–15 (10.0 ± 2.4 years); M:F = 25:10; without medication; without comorbidities. **Group 2 (HC):** n = 35; age 7–15 (10.2 ± 2.9 years); M:F = 23:12; screened for psychopathology.	*Plasma* - ↑ MDA, NO pool (NO_2-_, NO_3-_)	
**Doneray, 2022 [54]**	**Group 1 (ADHD):** n = 30; age 6–12 (10.03 ± 1.61 years); M:F = 18:12; without medication; without other comorbidities besides ODD. **Group 2 (HC):** n = 30; age 6–12 (9.87 ± 0.97 years); M:F = 16:14; screened for psychopathology.	*Serum* - ↑ arginine, ADMA	- no significant correlations with parameters of ADHD severity
**Elhady, 2019 [55]**	**Group 1 (epilepsy):** n = 25; age 7–14 (9.32 ± 0.90 years); M:F = 14:11; without comorbidities. **Group 2 (epilepsy + ADHD):** n = 25; age 7–14 (9.77 ± 1.63 years); M:F = 18:7; some of them received treatment; without comorbidities. **Group 3 (HC):** n = 35; age 7–14 (9.83 ± 1.52 years); M:F = 18:17; screened for psychopathology.	*Serum* - ↑ MDA, especially in those with ADHD (after logistic regression, MDA was the main predictor for ADHD in children with epilepsy)	
**Guney, 2015 [56]**	**Group 1 (ADHD):** n = 52; age range not specified (9.28 ± 2.78 years); M:F = 43:13; without medication; without comorbidities. **Group 2 (HC):** n = 52; age range not specified (9.69 ± 2.55 years); M:F = 36:16; screened for psychopathology.	*Plasma* - ↓ total thiols	
**Jansen, 2020 [57]**	**Group 1 (ADHD):** n = 42; age 6–16 (mean age not specified per entire group); M:F = 33:9; many of them received treatment; no data about comorbidities. **Group 2 (HC):** n = 43; age range not specified (9.7 ± 2.6 years); M:F = 28:15; not mentioned whether they were screened for psychopathology.	*Plasma* - ↑ NO_2-_, NO_3-_ - ↓ ADMA - *in patients with treatment:* ↑ NO_2-_ in comparison to those untreated *Urine* - no statistically significant differences	
**Nasim, 2019 [58]**	**Group 1 (ADHD):** n = 36; age 6–13 (9.4 ± 1.6 years); M:F ratio not specified; most of them received treatment; no data about comorbidities. **Group 2 (HC):** n = 32; age range not specified (9.5 ± 1.9 years); M:F ratio not specified; not screened for psychopathology.	*Serum* - ↓ GSH - no statistically significant differences in MDA levels	
**Öğütlü, 2020 [59]**	**Group 1 (ADHD):** n = 50; age 6–16 (median: 10 years); M:F = 33:17; without medication; without comorbidities. **Group 2 (HC):** n = 47; age 6–16 (median age: 9); M:F = 22:25; screened for psychopathology.	*Plasma* - ↓ native thiols, total thiols, disulfide - ↓ disulfide/native thiols ratio, disulfide/total thiols ratio - ↑ native thiols/total thiols index	- native thiols, total thiols = negatively correlated with inattention and hyperactivity - total thiols = negatively correlated with ADHD index
**Oztop, 2012 [60]**	**Group 1 (ADHD):** n = 30; age 6–12 (8.7 ± 1.91 years); M:F = 27:3; without medication; without comorbidities. **Group 2 (HC):** n = 30; age 6–12 (9.1 ± 2.09 years); M:F = 18:12; not screened for psychopathology.	*Serum* - ↓ MDA, 8-OH-dG *Plasma* - no statistically significant differences in thiol levels	- no significant correlations with parameters of ADHD severity
**Simsek, 2016 [61]**	**Group 1 (ADHD):** n = 25; age 6–10 (median: 8); M:F = 18:7; without medication; without comorbidities. **Group 2 (ADHD + DBD):** n = 24; age 7–11.3 (median: 8); M:F = 23:1; without medication; without other comorbidities. **Group 3 (HC):** n = 40; age 7–10 (median: 8); M:F = 32:8; screened for psychopathology.	*Serum* - no statistically significant differences in 8-OH-dG	- no significant correlations with parameters of ADHD severity
**Verlaet, 2019 [62]**	**Group 1 (ADHD):** n = 57; age 6–12 (8.98 ± 1.75 years); M:F = 41:16; without medication; “no diagnosis of ASD or severe mental conditions” **Group 2 (HC):** n = 69; age 6–12 (8.37 ± 1.69 years); M:F = 45:24; “no diagnosis of ASD or severe mental conditions”.	*Plasma* - no statistically significant differences in MDA after corrections *RBC* - ↑ GSH *Urine* - no statistically significant differences in 8-OH-dG	- weak positive correlation between plasma MDA and impulsivity
**Lipid metabolism**			
**Bekaroğlu, 1996 [63]**	**Group 1 (ADHD):** n = 48; age 6.5–12 (9.2 ± 2 years); M:F = 33:15; no data about medication or comorbidities. **Group 2 (HC):** n = 45; age 6.5–12 (9.3 ± 2 years); M:F = 30:15; not mentioned whether they were screened for psychopathology.	*Serum* - ↓ free FA	
**Chen, 2004 [64]**	**Group 1 (ADHD):** n = 58; age 4–12 (8.5 ± 2.2 years); M:F = 53:15; some of them received medication; no data about comorbidities. **Group 2 (HC):** n = 52; age range not specified (7.9 ± 2.0 years); M:F = 40:12; not mentioned whether they were screened for psychopathology.	*Plasma* - ↑ GLA - no statistically significant differences in AA, EPA, DHA, n-3, n-6, or other parameters *RBC membrane* - ↑ oleic acid, n-6/n-3 ratio - ↓ AA, DHA, nervonic acid, LA, total n-3 - no statistically significant differences in EPA, total n-6	
**Colter, 2008 [65]**	**Group 1 (ADHD):** n = 11; age 10.4–16.4 (13.6 ± 2.2 years); M:F = 9:2; most of them received medication; some of them had comorbid learning disorder. **Group 2 (HC):** n = 12; age 11.3–16.6 (14.2 ± 1.9 years); M:F = 6:6; not mentioned whether they were screened for psychopathology.	*RBC membrane* - ↓ DHA, n-3/n-6 ratio, total n-3 - no statistically significant differences in AA, ALA, EPA, LA, PUFA, n-6, or other parameters	- DHA = negatively correlated with oppositional behavior, hyperactivity, cognitive problems, restlessness, problematic behavior, inattention, and DSM-IV total score - total n-3 = negatively correlated with restlessness - n-3: n-6 ratio = negatively correlated with oppositional, restless, and problematic behavior - total n-6 = positively correlated with opposition, restlessness, problematic behavior, inattention, DSM-IV total, and ADHD index scales.
**Crippa, 2018 [66]**	**Group 1 (ADHD):** n = 51; age 7–14 (11.0 ± 1.6 years); M:F = 47:4; no data about medication or comorbidities. **Group 2 (HC):** n = 22; age 7–14 (11.4 ± 1.9 years); M:F = 22:1; screened for psychopathology.	*Whole blood* - ↓ DHA, n-3, PUFA - no statistically significant differences in AA, AA/DHA, AA/EPA, EPA, LA, MUFA, or saturated FA	- PUFA (especially DHA, EPA, and their sum) = negatively correlated with parent-reported symptoms - PUFA = negatively correlated with quality of life - AA/DHA ratio, MUFA, SFA = positively correlated with severity
**Gow, 2013 [67]**	**Group 1 (ADHD):** n = 29; age 12–16 (14.08 ± 1.45 years); only male participants; many of them received medication; without other comorbidities besides CD/ODD **Group 2 (HC):** n = 43; age range not specified (13.79 ± 2.23 years); only male participants; screened for psychopathology.	*Plasma* - no statistically significant differences in AA, DHA, EPA, n-3, n-6, or other parameters after correction	- EPA, n-3 = negatively correlated with callous/unemotional traits - no correlations between long-chain PUFA and aggressiveness, impulsivity, or self-concept
**Grazioli, 2019 [68]**	**Group 1 (ADHD):** n = 24; age range 8–14 (11.5 ± 1.5 years); M:F = 24:0; without medication; without comorbidities. **Group 2 (HC):** n = 21; age range not specified (11.3 ± 1.8 years); M:F = 20:1; screened for psychopathology.	*Whole blood* - ↓ AA - no statistically significant differences in DHA, EPA, LA	- EPA = negatively correlated with oxyhemoglobin in right fronto-pariental brain regions during a 0-back task (assessing working memory)
**Henríquez-Henríquez, 2015a [69]**	**Group 1 (ADHD):** n = 27; age 5–18 (mean age not specified); gender distribution not specified; no data regarding medication or comorbidities. **Group 2 (healthy first-degree relatives of ADHD patients):** n = 27; age not specified; included adults; gender distribution not specified; not mentioned whether they were screened for psychopathology. **Group 3 (HC):** n = 18; age not specified; included adults; gender distribution not specified; not mentioned whether they were screened for psychopathology.	*Whole plasma* - only in females, ↑ ALA, DPA, DHA/ALA, n-6 - only in females, ↓ DHA/DPA, n-3/n-6 ratio - only in males, ↓ ALA - no statistically significant differences in AA, DHA, EPA, LA, or n-3	- n-3/PUFA ratio = positively correlated with clinical status
**Henríquez-Henríquez, 2015b [70]**	**Group 1 (ADHD):** n = 28; age 5–18 (median: 12.8 years); M:F = 14:14; all of them received treatment (metylphenidate or D-amphetamine); no data regarding comorbidities. **Group 2 (healthy first-degree relatives of ADHD patients):** n = 28; median age: 34 years (included adults); M:F = 4:24; not mentioned if they were screened for psychopathology. **Group 3 (HC):** n = 21; median age: 23.5 years (included adults); M:F = 8:13; not mentioned if they were screened for psychopathology.	*Serum* - ↓ all assayed sphingomyelins (especially C24:1), except for C18:1, between ADHD and both control groups - ↓ ceramide C24:0, deoxyceramide C24:1 - no other statistically significant differences	
**Kozielec-Oracka, 2022 [71]**	**Group 1 (High-functioning autism and Asperger’s syndrome):** n = 33 (not clearly stated); age range not specified (10.4 ± 2.9 years); M:F = 39:5; without medication; no data regarding comorbidities other than “no comorbid severe conditions and illness”. **Group 2 (Healthy controls, siblings of patients):** n = 17; age range not specified (11.6 ± 3.3 years); M:F = 7:10; no data regarding comorbidities other than “no comorbid severe conditions and illness”.		- DHA, EPA phosphatidylcholines extracted from plasma phospholipids, and total n-3 = positively correlated with ADHD index - EPA phosphatidylcholine, EPA and DHA phosphatidylethanolamines from RBC, and total n-3 = negatively correlated with ADHD index - plasma DHA phosphatidylcholine and total n-3 = negatively correlated with inattention
**Miklavcic, 2023 [72]**	**Group 1 (ADHD):** n = 103; age 5–12 (8.23 ± 1.9 years); M:F = 90:13; most of them received medication; patients with comorbidities were included. **Group 2 (HC):** n = 26; age 5–12 (6.50 ± 0.8 years); M:F = 14:12; not mentioned whether they were screened for psychopathology.	*Plasma phospholipids* - ↓ AA, DHA	- DHA = negatively correlated with inattention
**Mitchell, 1987 [73]**	**Group 1 (ADHD):** n = 44; age range not specified (9.1 ± 2.3 years); gender distribution not specified; one patient received medication; no data regarding comorbidities. **Group 2 (HC):** n = 45; age range not specified (8.7 ± 2.3 years); gender distribution not specified; not mentioned whether they were screened for psychopathology.	*Serum phospholipids* - ↓ AA, DGLA, DHA - no statistically significant differences in EPA	- AA = negatively correlated with speech difficulties, learning difficulties, and delayed development
**Parletta, 2016 [74]**	**Group 1 (ADHD):** n = 401; age 3–17 (9.10 ± 3.58 years); M:F = 319:82; many of them received medication; no data regarding comorbidities. **Group 2 (ASD):** n = 85; age 3–17 (5.31 ± 2.12 years); M:F = 68:17; without medication; no data regarding comorbidities. **Group 3 (HC):** n = 79; age 3–17 (8.32 ± 2.53 years); M:F = 61:18; screened for psychopathology.	*RBC phospholipids* - ↓ AA, DHA, EPA, n-3/n-6 ratio - ↑ AA/EPA ratio	- AA, DHA, EPA, n-3/n-6 ratio = positively correlated with attention and impulsivity - AA/EPA ratio = negatively correlated with attention and impulsivity
**Spahis, 2008 [75]**	**Group 1 (ADHD):** n = 37; age 6–12 (9.01 ± 1.62 years); M:F = 27:10; no data regarding medication; without comorbidities. **Group 2 (HC):** n = 35; age 6–12 (8.53 ± 2.08 years); gender distribution not specified; screened for psychopathology.	*RBC phospholipids* - ↑ DHA, EPA, n-3 - ↓ ALA, oleic acid, n-6/n-3 ratio - no statistically significant differences in AA, n-6, or other parameters *Whole plasma* - ↑ DHA, EPA, palmitic acid - no statistically significant differences in AA, n-3, n-6, n-6/n-3 ratio, or other parameters - ↓ MDA	
**Stevens, 1995 [76]**	**Group 1 (ADHD):** n = 53; age 6–12 (9.1 ± 2 years); only male participants; many of them received treatment; no data regarding comorbidities. **Group 2 (HC):** n = 43; age 6–12 (9.1 ± 2.3 years); only male participants; not mentioned whether they were screened for psychopathology.	*Plasma polar lipids* - ↓ AA, DHA, EPA - ↑ n-3, n-6/n-3 ratio - no statistically significant differences in n-6 *RBC* - ↓ AA - no statistically significant differences in DHA (reported as significant, but with a *p* < 0.06), EPA, n-3, n-6, n-6/n-3	
**Stevens, 1996 [77]**	**ADHD group:** n = 96; age 6–12 (mean age not specified); only male participants; some of them (n = unspecified) had ADHD. **No control group.** Instead, the ADHD patients were grouped based on n-3 and n-6 concentrations in plasma phospholipids, with no age differences between groups.		- n-3 = negatively correlated with several ADHD scores, including hyperactivity and impulsivity
**Wang 2019 [78]**	**Group 1 (ADHD):** n = 216; age range not specified (9.2 ± 1.7 years); M:F = 186:30; no data regarding medication or comorbidities. **Group 2 (HC):** n = 216; age range not specified (9.2 ± 1.8 years); M:F = 186:30; not mentioned whether they were screened for other disorders.	*Serum* - ↑ PUFA, n-6/n-3 ratio	
**Yonezawa, 2018 [79]**	**ADHD group:** n = 24; age 9–19 (13.4 ± 3. years); M:F = 19:5; all of them received medication (methylphenidate or atomoxetine); no data regarding comorbidities. **No control group.** Instead, reference values for healthy adults were used for comparisons.	*Whole plasma* - ↓ AA, DHA, EPA, EPA/AA ratio	
**Amino acids metabolism**			
**Altun, 2018 [80]**	**Group 1 (ADHD):** n = 30; age 6–15 (9.3 ± 1.8 years); M:F = 23:7; without medication; without comorbidities. **Group 2 (HC):** n = 30; age 6–15 (9.46 ± 1.87 years); M:F = 21:9; screened for psychopathology.	*Serum* - ↓ homocysteine	- no significant correlations with ADHD symptoms or severity
**Bornstein, 1990 [81]**	**Group 1 (ADHD):** n = 28; age range not specified (9.6 ± 2.4 years); M:F = 23:5; without medication; without comorbidities. **Group 2 (HC):** n = 20; age range not specified (10.8 ± 3.8 years); M:F = 8:12; screened for psychopathology.	*Plasma* - ↓ His, Iso, Tyr, Trp, Phe - no statistically significant differences in Ala, Val *Urine* - no statistically significant differences in Phe, Tyr	
**Bergwerff, 2016 [82]**	**Group 1 (ADHD):** n = 83; age 6–13 (9.72 ± 1.65 years); M:F = 62:21; most of them received medication; some of them had comorbidities. **Group 2 (HC):** n = 72; age 6–13 (9.93 ± 1.72 years); M:F = 37:35; screened for psychopathology.	*Plasma* - no statistically significant differences in Phe, Trp, Tyr *Urine* - no statistically significant differences in Phe, Trp, Tyr	- no significant correlations with ADHD symptoms or severity
**Hasan, 2016 [83]**	**ADHD group:** n = 100; age 2–12 (mean: 7.41 years); M:F = 75:25; no data regarding medication; excluded some comorbidities (anxiety, ASD, psychotic disorders). **No control group.** Instead, reference values were used for comparisons.	*Plasma* - ↑ ammonia, lactate	
**Hubers, 2024 [84]**	**Group 1 (ADHD):** n = 37; age 6–12.2 (9.5 ± 1.9 years); M:F = 23:14; no data regarding medication or comorbidities. **Group 2 (HC):** n = 221; age 5.7–12.7 (9.6 ± 2 years); M:F = 108:113; not screened for other disorders.		- three correlational patterns; one of them included some CpGs in the STAP2 gene, the transmitted ADHD polygenic risk, the transmitted self-reported health PGS, and 11 amino acids
**Liu, 2001 [85]**	**Group 1 (ADHD):** n = 20; age range not specified (mean: 9.4 ± 4.6 years); gender distribution not specified; no data regarding medication or comorbidities. **Group 2 (HC):** n = 140; age 1–13 (8.7 ± 4 years); gender distribution not specified; not mentioned whether they were screened for psychopathology.	*Urine* - identified a urinary tetrapeptide, Gly-Ser-Glu-Asn, which was present only in children with ADHD and in 2 autistic children, not in the HC group.	
**Rucklidge, 2019 [86]**	**ADHD group:** n = 71; age 7–12.9 (9.7 ± 1.5 years); M:F = 55:16; without medication at the beginning of the trial; some of them had comorbidities. **No control group.** Instead, reference values were used for comparisons.	*Serum* - no statistically significant differences in homocysteine	
**Skalny, 2021 [87]**	**Group 1 (ADHD):** n = 71; age 7–14 (8.4 ± 2.6 years); M:F = 54:17; without medication; no data regarding comorbidities. **Group 2 (HC):** n = 31; age 7–14 (8 ± 2.9 years); M:F = 24:7; not screened for psychopathology.	*Serum* - ↓ Glu, Pro, Gln/Glu ratio - ↑ Glu, hydroxyproline - no statistically significant differences in other amino acids (His, Iso, Leu, Lys, Met, Phe, Thr, Trp, Val)	- Gln, Lys = negatively correlated with total ADHD score - Glu = positively correlated with total ADHD score
**Wang, 2021b [88]**	**Group 1 (ADHD):** n = 31; age 6–16 (10.4 ± 2.2 years); M:F = 20:11; without medication; without comorbidities. **Group 2 (HC):** n = 29; age 6–16 (10.3 ± 2.9); M:F = 15:14; not screened for psychopathology.	*Plasma* - ↑ L-cystine	- only in children with ADHD, L-cystine = negatively correlated with decreased fMRI functional connectivity between regions of default mode network and regions influenced by the salience network
**Yektaș, 2019 [89]**	**Group 1 (ADHD):** n = 48; age range not specified (median: 9 years); M:F = 48:0; without medication **Group 2 (ASD):** n = 35; age range not specified (median: 8.6 years); M:F = 24:11; without medication **Group 3 (HC):** n = 35; age range not specified (median: 6 years); M:F = 28:7; not screened for psychopathology.	*Serum* - ↑ homocysteine	- homocysteine = positively correlated with hyperactivity and impulsivity
**Kynurenine pathway**			
**Dolina, 2014 [90]**	**Group 1 (ADHD, untreated):** n = 13; age 6–11 (mean not specified); gender distribution not specified; without medication; not mentioned whether they were screened for comorbidities. **Group 2 (ADHD, treated):** n = 10; age 6–11 (mean not specified); gender distribution not specified; treated with methylphenidate; not mentioned whether they were screened for comorbidities. **Group 3 (HC):** n = 41; age 6–11 (mean not specified); gender distribution not specified; not mentioned whether they were screened for psychopathology.	*Urine* - ↑ Trp, KYN, 3-OH-KYN *(only in those untreated)*, KA *(only in those untreated)* - ↓ indole sulfate, 3-OH-AA/3-OH-KYN ratio, 4-PA/Trp ratio, indole sulfate/Trp ratio, indole sulfate/KYN ratio	
**Evangelisti, 2017 [91]**	**Group 1 (ADHD):** n = 102; age range not specified (mean: 9.3 ± 2.7 years); M:F = 75:27; without medication; many of them had comorbidities. **Group 2 (HC):** n = 62; age range not specified (mean: 9.6 ± 1.74 years); M:F = 48:14; screened for psychopathology.	*Serum* - ↑ Trp, KYN, KYN/Trp ratio - ↓ anthranilic acid, KA, xanthurenic acid - no statistically significant differences in quinolinic acid, 3-OH-AA	- KA = negatively correlated with hyperactivity and total ADHD scores - KYN = positively correlated with inattention - presence of ADHD was predicted by ↓ anthranilic acid (AUC = 0.88) and ↑ Trp
**Hoshino, 1985 [92]**	**Group 1 (ADHD):** n = 10; age 5–10 (mean: 7.4 years); M:F = 5:5; no data regarding medication or comorbidities. **Group 2 (HC):** n = 12; age 7–14 (mean: 10.5); M:F = 6:6; not screened for psychopathology.	*Plasma* - no statistically significant differences in total Trp - ↑ free Trp, free Trp/total Trp ratio	- free Trp = positively correlated with severity
**Kilany, 2022 [93]**	**Group 1 (Learning disorder):** n = 69; age 6–13.4 (8.5 ± 1.6 years); M:F = 47:22; without medication; no data regarding other comorbidities. **Group 2 (Learning disorder + ADHD):** n = 31; age 6–12 (8.5 ± 1.8 years); M:F = 20:11; without medication; no data regarding other comorbidities. **Group 3 (HC):** n = 54; age 6–12 (8.5 ± 1.8 years); M:F = 35:19	*Plasma* - no statistically significant differences in KYN or MDA between patients with learning disorders comorbid with ADHD and children with learning disorders not comorbid with ADHD - ↑ KYN, MDA in children with learning disorders (±ADHD) and HC group	
**Molina-Carballo, 2021 [94]**	**Group 1 (ADHD):** n = 130; age 5–14 (9.47 ± 2.52 years); M:F = 102:28; without medication at the beginning of the trial; the group was further subclassified in two subgroups: (1) predominantly attention deficit (n = 52); (2) predominantly hyperactive–impulsive with comorbid CD (n = 78); without any other comorbidities. **Group 2 (HC):** n = 49 (most of them, n = 35, were siblings of the ADHD children); age 5–14 (10.35 ± 2.55 years); M:F = 33:16; screened for psychopathology.	*Plasma and urine* - no statistically significant differences in Trp, KYN, xanthurenic acid, anthranilic acid, quinolinic acid, or nicotinamide	
**Oades, 2010a [95]**	**Group 1 (ADHD, untreated):** n = 21; age 6.6–11.7 (8.84 ± 1.14 years); M:F = 14:7; without medication; some comorbidities were excluded (ASD, bipolar disorder, and others). **Group 2 (ADHD, treated):** n = 14; age 7.9–15.5 (12.6 ± 2.1 years); M:F = 12:2; treated with methylphenidate or atomoxetine; some comorbidities were excluded (ASD, bipolar disorder, and others). **Group 3 (HC):** n = 21; age 7.7–13.4 (11 ± 1.5 years); M:F = 20:1; screened for psychopathology. **Group 4 (Siblings of treated ADHD patients):** n = 7; age 9–14.4 (11.7 ± 2.1 years); M:F = 4:3; screened for psychopathology.	*Serum* - ↓ 3-OH-KYN - no statistically significant differences in levels of kynurenine and its other metabolites - ↑ Iso, Cys, Met, Pro - no statistically significant differences in Leu, Val - no statistically significant differences in 5-HIAA	
**Oades, 2010b [96]**	**The first three groups from above.**		- metabolites of Trp were not correlated with symptoms - ↑ Trp predicts omission errors - KYN = positively correlated with reaction time
**Sağlam, 2021 [97]**	**Group 1 (ADHD):** n = 46; mean age: 10.93 ± 2.54 years; M:F = 34:12. **Group 2 (ADHD + ODD):** n = 43; mean age: 10.79 ± 2.57 years; M:F = 31:12. **Group 3 (ADHD + CD):** n = 33; mean age: 11.70 ± 2.51; M:F = 29:4. **Group 4 (HC):** n = 50; mean age: 11.34 ± 2.7 years; M:F = 35:15; screened for psychopathology. All groups included participants of ages between 8 and 18 years, without medication. Some comorbidities were also excluded (ASD, bipolar disorder, schizophrenia, substance addiction).	*Serum* - *only in group 1:* ↑ KYN - *only in groups 1 and 2:* ↓ 3-OH-KYN - no statistically significant differences in Trp, KA, 3-OH-AA, Kyn/Trp ratio, or KA/3-OH-KYN	- KA = positively correlated with anxiety scores
**Neurotransmitters metabolism**			
**Baker, 1991 [98]**	**Group 1 (ADHD):** n = 18; age range not specified (9.6 ± 2.4 years); gender distribution not specified, but it is mentioned that there were more boys than girls in this group, as opposed to more girls than boys in Group 2; without medication for at least 2 weeks; without comorbidities. **Group 2 (HC):** n = 26; age range not specified (10.8 ± 3.8 years); gender distribution not specified, F > M; screened for psychopathology.	*Urine* - no statistically significant differences in PAA, Phe *Plasma* - ↓ Phe, Tyr - no statistically significant differences in PAA	
**Baker, 1993 [99]**	**Group 1 (ADHD):** n = 26; age range not specified (9.5 ± 2.4 years); M:F = 21:5; no data regarding medication or comorbidities. **Group 2 (HC):** n = 27; age range not specified (10.4 ± 3.4 years); M:F = 12:15; screened for psychopathology, although not specified how.	*Urine* - no statistically significant differences in MHPG, NME	
**Chatterjee, 2022 [100]**	**Group 1 (ADHD):** n = 35 (selected from a sample of 274 children, with an average age of 8.82 ± 3.24 years and an M/F ratio of 10/1); the rule of selection is not specified; no data regarding age or gender distribution; without medication; no data regarding comorbidities. **Group 2 (HC):** n = 26 (selected from a sample of 367 children, with an average age of 9.97 ± 5.4 years and a M/F ratio of 10/3); the rule of selection is not specified; no data regarding age or gender distribution, although it is mentioned that they were age-matched; screened for psychopathology.	*Plasma* - ↓ 5-HIAA (especially in presence of the 5-HTTLPR S/S genotype as compared to the L/L or L/S genotypes)	
**Hanna, 1996 [101]**	**Group 1 (ADHD):** n = 15; age 7–11 (9.25 ± 1.32 years); only male; without medication in the last 2 weeks; without comorbidities. **Group 2 (HC):** n = 16; age 7–11 (8.42 ± 1.36 years); only male; screened for psychopathology.	*Urine* - ↓ DOPEG - no statistically significant differences in DOPAC, DHPG	- DOPEG = negatively correlated with a broad range of behavioral measures, including number of ADHD symptoms, inattention, aggressivity, internalizing and externalizing scores.
**Khan, 1981 [102]**	**Group 1 (ADHD):** n = 10; age 6–11 (mean: 8.7 years); only male; no medication in the last 3 weeks; no data regarding comorbidities. **Group 2 (HC):** n = 10; age 6–11 (mean: 8.8 years); only male; were not screened for psychopathology.	*Urine (24 h)* - ↑ MHPG - no statistically significant differences in metanephrine or NME	
**Konrad, 2003 [103]**	**Group 1 (ADHD):** n = 31; age 8–12 (10.5 ± 1.6 years); M:F = 28:3; without medication; some of them had comorbidities (anxiety, ODD, CD). **Group 2 (Traumatic brain injuries):** n = 27; age 8–12 (10.6 ± 1.7 years); M:F = 19:8; some of them had mental disorders (anxiety, ODD, CD). **Group 3 (HC):** n = 26; age 8–12 (10.2 ± 1.2 years); M:F = 20:6; some of them had mental disorders (anxiety, ODD, CD).	*Urine* - ↑ NME - ↓ metanephrine post-stress	- no significant correlations of metabolites with ADHD symptoms or severity
**Moriarty, 2011 [104]**	**Group 1 (ADHD):** n = 17; no specifics given. **Group 2 (Control):** n = 20; no specifics given, only that they were “matched”	*Urine* - ↓ 5-HIAA	
**Oades, 1998 [105]**	**Group 1 (ADHD):** n = 14; age 6.5–14.3 (mean: 9.8 years); M:F = 13:1; without medication; included some comorbidities (CD, social and emotional disturbance, enuresis–encopresis, speech or motor developmental disorder). **Group 2 (HC):** n = 9; age 8.9–12.1 (mean: 10.6 years); M:F = 5:4; not mentioned whether they were screened for psychopathology.	*Urine* - ↑ 5-HIAA - no statistically significant differences in homovanillic acid, MHPG	
**Roessner, 2007 [106]**	**Group 1 (ADHD):** n = 42; age range not specified (12.1 ± 3.2 years); gender distribution not specified; many of them received medication; several comorbidities were included (CD, learning disorders, and tic disorders, while some of them were not specified). **Group 2 (HC):** n = 24; age range not specified, but included adults (23.8 ± 17 years); gender distribution not specified.	*Urine* - ↑ four free tetrahydroisoquinoline derivatives: salsolinol, N-methyl-salsolinol, norsalsolinol, N-methyl-norsalsolinol - ↑ conjugated norsalsolinol - ↑ total norsalsolinol, N-methyl-salsolinol	- free N-methyl-salsolinol = detects ADHD with 92.5% sensitivity and 94.4% specificity
**Shekim, 1987 [107]**	**Group 1 (ADHD):** n = 28; age 7–13.5 years (mean: 9.8 years); only male; without medication for at least 2 weeks; no data regarding comorbidities. **Group 2 (HC):** n = 23; age range not specified (9.9 ± 2 years); only male; not mentioned whether they were screened for psychopathology.	*Urine* - ↓ homovanillic acid, MHPG	
**Other metabolic processes**			
**Büber, 2016 [108]**	**Group 1 (ADHD):** n = 27; age 6–15 (9.37 ± 2.69); M:F = 23:4; without medication; without comorbidities. **Group 2 (HC):** n = 28; age 7–16 (10.5 ± 2.71); M:F = 21:7; screened for psychopathology.	*Urine* - ↑ 6-OH-MS (daytime, nighttime, 24-h)	- no significant correlations with ADHD symptoms or severity
**Fernández-López, 2020 [109]**	**Group 1 (ADHD):** n = 107; age range not specified (9.47 ± 2.52 years); gender distribution not specified; without medication; main comorbidities were excluded. **Group 2 (HC):** n = 41 (mainly siblings of patients with ADHD, n = 35); age range not specified (10.35 ± 2.55 years); gender distribution not specified; not mentioned whether they were screened for psychopathology.	*Serum and urine* - no statistically significant differences in tryptamine, indoleacetic acid, indolepropionic acid	
**Molina-Carballo, 2013 [110]**	**Group 1 (ADHD):** n = 136; age 5–14 (9.45 ± 2.52 years); M:F = 106:30; some of them received medication; no data regarding comorbidities. **Group 2 (HC):** n = 42 (including siblings); age 5–14 (10.35 ± 2.55 years); M:F = 30:12; not mentioned whether they were screened for psychopathology.	*Urine* - no statistically significant differences in 6-S-aMT	
**Sari, 2020 [111]**	**Group 1 (ADHD):** n = 35; age 8–11 (mean: 9 years); M:F = 27:8; without medication; without comorbidities. **Group 2 (HC):** n = 35; age 9–14 (mean: 10 years); M:F = 26:9; screened for psychopathology.	*Serum* - ↑ agmatine, Arg - no statistically significant differences in Glu	- in those with ADHD, moderate positive correlation between Arg and Glu with NO levels
**Untargeted metabolomics**			
**Swann, 2023 [112]**	**Group 1 (ADHD):** n = 33; age 8–19 (13.55 ± 3.01 years); M:F = 19:14; no data regarding medication or comorbidities. **Group 2 (HC):** n = 79; age 8–22 (15.17 ± 2.99 years); M:F = 42:37; screened for psychopathology. All participants were included from an ongoing twin study.	*Urine* - only in males, ↑ hippurate	
**Tian, 2022 [113]**	**Group 1 (ADHD without tic disorders):** n = 44; age range not specified (7.9 ± 2 years); M:F = 38:6; without medication or comorbidities. **Group 2 (ADHD with tic disorders):** n = 32; age range not specified (8.7 ± 1.8 years); M:F = 28:4; without medication or other disorders. **Group 3 (HC):** n = 63; age range not specified (7.8 ± 1.8 years); M:F = 58:5; screened for psychopathology.	*Urine* - 34 metabolites which distinguished between pure ADHD and HC	- a metabolite panel of FAPy-adenine, 3-methylazelaic acid, and phenylacetylglutamine predicted ADHD with AUC = 0.918
**Wang, 2021a [114]**	**Group 1 (ADHD):** n = 58; age range not specified (9 ± 2.3 years); M:F = 45:13; no data regarding medication or comorbidities. **Group 2 (HC):** n = 38; age range not specified (10.2 ± 2.9 years); M:F = 21:17; screened for psychopathology.	*Plasma* - ↑ guanosine, o-phosphoethanolamine, phenyl-leucine, hypoxanthine, 4-aminohippuric acid, 5-hydroxylysine, L-cystine - ↓ gentisic acid, tryptophyl-phenylalanine	- o-phosphoethanolamine, 4-aminohippuric acid, 5-hydroxylysine, L-cystine, tryptophyl-phenylalanine, gentisic acid = correlated with ADHD symptoms

Legend: AA = arachidonic acid; ADMA = asymmetric dimethylarginine; Ala = alanine; ALA = α-linolenic acid; Arg = arginine; ASD = autism spectrum disorder; CD = conduct disorder; Cys = cystine; DBD = disruptive behavioral disorders; DGLA = dihomo-γ-linolenic acid; DHA = docosahexaenoic acid; DHPG = 3,4-dihydroxyphenyl glycol; DOPAC = 3,4-dihydroxyphenylacetic acid; DOPEG = dihydroxyphenyletylene glycol; DPA = docosapentaenoic acid; EPA = eicosapentaenoic acid; FA = fatty acids; GLA = γ-linolenic acid; Gln = glutamate; Glu = glutamine; GSH = glutathione; HC = healthy controls; His = histidine; Iso = isoleucine; KA = kynurenic acid; KYN = kynurenine; LA = linoleic acid; Leu = leucine; Lys = lysine; MDA = malondialdehyde; Met = methionine; MHPG = 3-methoxy-4-hydroxyphenylethylene glycol; MUFA = monounsaturated fatty acids; n-3 = omega-3; n-6 = omega-6; NME = normetanephrine; NO = nitric oxide; NO_2-_ = nitrites; NO_3-_ = nitrates; ODD = oppositional defiant disorder; PAA = phenylacetic acid; Phe = phenylalanine; Pro = proline; PUFA = polyunsaturated fatty acids; RBC = red blood cells; SFA = saturated fatty acids; Trp = tryptophan; Tyr = tyrosine; Val = valine; 3-OH-AA = 3-hydroxyanthranilic acid; 3-OH-KYN = 3-hydroxykynurenine; 4-PA = 4-pyridoxic acid; 5-HIAA = 5-hydroxyindoleacetic acid; 6-OH-MS = 6-hydroxymelatonin sulfate; 6-S-aMT = 6-sulfatoxymelatonin; 8-OH-dG = 8-hydroxy-2′-deoxyguanosine.

**Table 2:** Main fatty acids for which significant differences have been identified.

	DHA	EPA	AA	n-3	n-6
Plasma	- ↓: **4 studies—**Miklavcic 2023, Mitchel 1987, Stevens 1995, Yonezawa 2018 - no difference: **3 studies—**Chen 2004, Gow 2013, Henríquez-Henríquez 2015a - ↑: **1 study—**Spahis 2008	- ↓: **2 studies—**Stevens 1995, Yonezawa 2018 - no difference: **4 studies**—Chen 2004, Gow 2013, Henríquez-Henríquez 2015a, Mitchel 1987 - ↑: **1 study—**Spahis 2008	- ↓: **4 studies—**Miklavcic 2023, Mitchel 1987, Stevens 1995, Yonezawa 2018 - no difference: **4 studies—**Chen 2004, Gow 2013, Henríquez-Henríquez 2015a, Spahis 2008 - ↑: **0 studies**	- ↓: **0 studies** - no difference: **4 studies—**Chen 2004, Gow 2013, Henríquez-Henríquez 2015a, Spahis 2008 - ↑: **1 study—**Stevens 1995	- ↓: **0 studies** - no difference: **5 studies—**Chen 2004, Gow 2013, Henríquez-Henríquez 2015a, Spahis 2008, Stevens 1995 - ↑: **0 studies**
Whole blood	- ↓: **1 study—**Crippa 2018 - no difference: **1 study**—Grazioli 2019 - ↑: **0 studies**	- ↓: **0 studies** - no difference: **2 studies—**Crippa 2018, Grazioli 2019 - ↑: **0 studies**	- ↓: **1 study—**Grazioli 2019 - no difference: **1 study**—Crippa 2018 - ↑: **0 studies**	- ↓: **0 studies** - no difference: **0 studies** - ↑: **1 study—**Crippa 2018	- ↓: **0 studies** - no difference: **0 studies** - ↑: **0 studies**
Red blood cell membrane	- ↓: **3 studies—**Chen 2004, Colter 2008, Parletta 2016 - no difference: **0 studies** - ↑: **1 study—**Spahis 2008	- ↓: **1 study—**Parletta 2016 - no difference: **2 studies—**Chen 2004, Colter 2008 - ↑: **1 study—**Spahis 2008	- ↓: **3 studies—**Chen 2004, Parletta 2016, Stevens 1996 - no difference: **0 studies** - ↑: **1 study—**Spahis 2008	- ↓: **2 studies—**Chen 2004, Colter 2008 - no difference: **1 study—**Stevens 1996 - ↑: **1 study—**Spahis 2008	- ↓: **0 studies** - no difference: **4 studies—**Chen 2004, Colter 2008, Spahis 2008, Stevens 1996 - ↑: **0 studies**

## Data Availability

No new data were created or analyzed in this study. Data sharing is not applicable to this article.

## Supplementary Materials

The supporting information can be downloaded at: https://www.mdpi.com/article/10.3390/ijms25084385/s1.
