# Supplementary material for: Metabolomic Markers in Attention-Deficit/Hyperactivity Disorder (ADHD) among Children and Adolescents—A Systematic Review
Source: Int J Mol Sci. 2024 Apr 16;25(8):4385. doi: 10.3390/ijms25084385 (PMC11050195; doi:10.3390/ijms25084385)
Supplement: Supplementary file 1 [file ijms-25-04385-s001.zip › Supplementary File S1_Search_strings.pdf]

<u>Supplementary File S 1:</u> Search strings adapted for each database.

- PubMed: ((metabolom*) OR (metabolit*) OR (omics) OR (plasma) OR (serum) OR (urin*)) AND ((adhd[Title/Abstract]) OR (hyperkine*[Title/Abstract]) OR (attention defic*[Title/Abstract])) AND ((children) OR (adolescent*)) **=> 1563 results**

- WoS: (TS=(metabolom*) OR TS=(metabolit*) OR TS=(omics) OR TS=(plasma) OR TS=(serum) OR TS=(urin*)) AND (TS=(ADHD) OR TS=(hyperkine*) OR TS=("attention defic"*)) AND (TS=(children) OR TS=(adolescent*)) **=> 2839 results**
