# Supplementary material for: Metabolomic Markers in Attention-Deficit/Hyperactivity Disorder (ADHD) among Children and Adolescents—A Systematic Review
Source: Int J Mol Sci. 2024 Apr 16;25(8):4385. doi: 10.3390/ijms25084385 (PMC11050195; doi:10.3390/ijms25084385)
Supplement: Supplementary file 1 [file ijms-25-04385-s001.zip › Supplementary Table S1_Newcastle-Ottawa Scale Rating..pdf]

Supplementary Table S 1: **Newcastle-Ottawa Scale Rating.**
Quality assessment of the included studies using the Newcastle-Ottawa Scale.

Study	SELECTION (max. 4 stars)								COMPARABILITY (max. 2 stars)				OUTCOME (max. 2 stars)				TOTAL (max. 8 stars)		Traffic Light plot
	Case definition		Representativeness of cases		Controls selection		Controls definition		Age, gender		Additional		Assessment		Statistical procedures				
	R1	R2	R1	R2	R1	R2	R1	R2	R1	R2	R1	R2	R1	R2	R1	R2	R1	R2	
**Oxidative stress**																			
Avcil, 2017	★	★	★	★		★	★	★	★	★	★	★	★	★	★	★	7	8	+ + + +
Avcil, 2021	★	★	★	★	★		★	★	★	★	★	★	★	★	★	★	8	7	+ + + +
Ceylan, 2010	★	★	★	★			★	★	★	★	★	★		★	★	★	6	7	+ + + +
Doneray, 2022	★	★	★	★	★		★	★	★	★	★		★	★	★	★	8	6	+ + + +
Elhady, 2019	★	★			★	★			★	★	★	★	★	★	★	★	6	6	- + + +
Guney, 2015	★	★	★	★			★	★	★	★			★	★	★	★	6	6	+ - + +
Jansen, 2020	★	★	★	★				★	★	★	★	★	★	★	★	★	6	7	- + + +
Nasim, 2019	★	★					★	★					★	★	★	★	4	4	- x + -
Öğütlü, 2020	★	★	★	★	★	★	★	★	★	★		★	★	★		★	6	8	+ + + +
Oztop, 2012	★	★	★			★	★	★	★		★	★	★	★	★	★	7	6	- + + +
Simsek, 2016	★	★	★	★	★		★	★	★	★	★	★	★	★	★	★	8	7	+ + + +
Verlaet, 2019	★	★	★	★		★			★	★	★	★	★	★	★	★	6	7	- + + +
**Lipid metabolism**																			
Bekaroğlu, 1996	★	★	★	★					★	★				★	★		4	4	- - - -
Chen, 2004	★	★	★	★	★	★			★	★	★		★	★	★	★	7	7	+ + + +
Colter, 2008	★	★		★	★	★		★	★	★	★		★	★		★	5	7	+ + + +

Crippa, 2018	★	★	★	★	★	★	★	★	★	★	★	★	★	★	★	★	8	8	+ + + +
Gow, 2013	★	★			★	★	★	★			★	★	★	★	★	★	6	7	+ - + +
Grazioli, 2019	★	★		★	★	★	★	★	★	★	★	★	★	★		★	6	8	+ + + +
Henríquez-Henríquez, 2015a	★	★				★		★			★		★	★	★	★	4	5	- x + -
Henríquez-Henríquez, 2015b	★	★				★		★				★	★	★	★		3	5	- x + -
Kozielec-Oracka, 2022											★	★		★			1	2	x - x x
Miklavcic, 2023	★	★								★	★		★	★	★		4	3	x - x -
Mitchell, 1987			★		★	★			★	★			★	★		★	4	4	- - + -
Parletta, 2016				★	★		★		★			★	★	★	★	★	5	4	- - + -
Spahis, 2008	★	★	★	★		★	★	★	★	★		★	★	★	★	★	6	8	+ + + +
Stevens, 1995	★	★			★	★			★		★		★	★	★	★	6	4	- - + -
Stevens, 1996		★				★			★				★	★	★	★	3	4	x x + -
Wang 2019b	★	★	★	★	★	★	★	★	★	★	★		★	★	★	★	8	7	+ + + +
Yonezawa, 2018	★	★	★										★	★	★		4	2	- x + -
**Aminoacids metabolism**																			
Altun, 2018	★	★	★	★	★	★	★	★	★	★			★	★	★	★	7	7	+ - + +
Bornstein, 1990	★	★	★	★		★	★	★	★			★			★	★	5	6	+ - - -
Bergwerff, 2016	★	★		★	★		★	★				★	★	★	★	★	5	6	+ - + -
Hasan, 2016		★	★											★	★		2	2	x x - x

Hubers, 2024	★	★	★	★					★	★	★	★	★	★	★	★	6	6	- + + +
Liu, 2001		★				★								★			0	3	x x x x
Rucklidge, 2019	★		★	★								★	★	★		★	3	4	- x + -
Skalny, 2021	★	★	★	★	★		★	★	★	★	★		★	★		★	7	6	+ + + +
Wang, 2021b	★	★	★	★	★	★		★	★	★	★		★	★	★	★	7	7	+ + + +
Yektas, 2019	★	★				★			★		★	★	★		★	★	5	4	- + + -
**Kinurenine pathway**																			
Dolina, 2014											★		★		★	★	3	1	x x + x
Evangelisti, 2017	★	★	★	★	★	★	★	★	★	★	★	★	★	★	★	★	8	8	+ + + +
Hoshino, 1985	★	★	★												★		3	1	- x x x
Kilany, 2022	★		★						★	★	★	★	★		★	★	6	3	x + + -
Molina-Carballo, 2021	★	★	★	★			★		★		★	★	★	★	★	★	7	5	+ + + +
Oades, 2010a	★	★	★	★	★	★	★	★	★	★			★	★	★	★	7	7	+ - + +
Oades, 2010b	★	★	★	★	★	★	★	★	★	★			★	★	★	★	7	7	+ - + +
Sağlam, 2021	★	★	★	★	★	★	★	★	★	★	★	★	★	★	★	★	8	8	+ + + +
**Neurotransmitters metabolism**																			
Baker, 1991	★	★	★			★	★	★	★				★	★			5	4	+ x - -
Baker, 1993	★	★	★			★	★	★	★	★			★	★			5	5	+ - - -
Chatterjee, 2022	★	★					★	★			★	★	★	★			4	4	- - - -
Hanna, 1996	★	★			★		★	★	★		★	★	★	★	★		7	4	- + + +
Khan, 1981	★	★				★		★	★				★				3	3	- x x -

Konrad, 2003	★	★		★	★	★	★	★	★	★	★	★	★	★	★	★	7	8	+ + + +
Moriarty, 2011													★	★			1	1	X X X X
Oades, 1998	★	★							★	★	★	★	★	★	★	★	5	5	X + + -
Roessner, 2007	★	★				★	★	★	★	★	★		★	★	★	★	6	6	- + + +
Shekim, 1987	★	★						★	★				★	★	★	★	4	4	- X + -
**Other metabolic processes**																			
Büber, 2016	★	★	★	★	★	★	★	★	★	★			★	★	★	★	7	7	+ - + +
Fernández-López, 2020	★	★	★	★							★	★	★	★	★	★	5	5	- - + -
Molina-Carballo, 2013	★	★	★	★				★	★				★	★	★	★	5	5	- X + -
Sari, 2020	★	★	★	★	★		★	★	★	★	★	★	★	★	★	★	8	7	+ + + +
**Untargeted metabolomics**																			
Swann, 2023	★	★	★	★		★	★	★	★	★	★	★	★	★	★	★	7	8	+ + + +
Tian, 2022	★	★	★	★	★	★	★	★	★	★	★	★	★	★	★	★	8	8	+ + + +
Wang, 2021a	★	★	★	★	★	★	★	★	★	★	★	★	★	★	★	★	8	8	+ + + +

Traffic light plot legend:

	Selection	Comparability	Outcome	Total
+	sum of stars 6-8	sum of stars 3-4	sum of stars 3-4	sum of stars 12-16
-	sum of stars 3-5	sum of stars 2	sum of stars 2	sum of stars 6-11
X	sum of stars 0-2	sum of stars 0-1	sum of stars 0-1	sum of stars 0-5
