# Supplementary material for: Metabolomic Markers in Attention-Deficit/Hyperactivity Disorder (ADHD) among Children and Adolescents—A Systematic Review
Source: Int J Mol Sci. 2024 Apr 16;25(8):4385. doi: 10.3390/ijms25084385 (PMC11050195; doi:10.3390/ijms25084385)
Supplement: Supplementary file 1 [file ijms-25-04385-s001.zip › Supplementary Table S2_Main_findings.pdf]

<u>Supplementary Table S 2:</u> Summary of the main findings.

Biomarker	↑	No difference	↓
**Oxidative stress**			
**MDA**	**2 studies:** Ceylan, 2010; Elhady, 2019	**4 studies:** Avcil, 2021; Kilany, 2022 (compared LD + ADHD vs. LD); Nasim, 2019; Verlaet, 2019	**2 studies:** Oztop, 2012; Spahis, 2008
**8-OHdG**		**2 studies:** Simsek, 2016; Verlaet, 2019(u)	**1 study:** Oztop, 2012
**Total thiols**	**1 study:** Avcil, 2017	**1 study:** Oztop, 2012	**2 studies:** Guney, 2015; Öğütlü, 2020
**NO pool**	**2 studies:** Ceylan, 2010; Jansen, 2020		
**ADMA**	**1 study:** Doneray, 2022		**1 study:** Jansen, 2020
**GSH**	**1 study:** Verlaet, 2019		**1 study:** Nasim, 2019
**Lipid metabolism** – selected markers; detailed in Table 2			
**DHA**	**1 study:** Spahis, 2008	**4 studies:** Chen, 2004; Gow, 2013; Grazioli, 2019; Henríquez-Henríquez, 2015a	**8 studies:** Chen, 2004; Colter, 2008; Crippa, 2018; Miklavcic, 2023; Mitchel, 1987; Parletta, 2016; Stevens, 1995; Yonezawa, 2018
**EPA**	**1 study:** Spahis, 2008	**7 studies:** Chen, 2004; Colter, 2008; Crippa, 2018; Gow, 2013; Grazioli, 2019; Henríquez-Henríquez, 2015a; Mitchel, 1987	**3 studies:** Parletta, 2016; Stevens, 1995; Yonezawa, 2018
**AA**	**1 study:** Spahis, 2008	**5 studies:** Chen, 2004; Crippa, 2018; Gow, 2013; Henríquez-Henríquez, 2015a; Spahis, 2008	**8 studies:** Chen, 2004; Grazioli, 2019; Miklavcic, 2023; Mitchel, 1987; Parletta, 2016; Stevens, 1995; Stevens, 1996; Yonezawa, 2018
**n-3**	**3 studies:** Crippa, 2018; Spahis, 2008; Stevens, 1995	**5 studies:** Chen, 2004; Gow, 2013; Henríquez-Henríquez, 2015a; Spahis, 2008; Stevens, 1996	**2 studies:** Chen, 2004; Colter, 2008
**n-6**	**0 studies**	**7 studies:** Chen, 2004; Colter, 2008; Gow, 2013; Henríquez-Henríquez, 2015a; Spahis, 2008; Stevens 1995; Stevens, 1996	**0 studies**
**Amino acids metabolism**			
**Homocysteine**	**1 study:** Yektaş, 2019	**1 study:** Rucklidge, 2019	**1 study:** Altun, 2018
**Phe, Tyr**		**2 studies:** Bergwerff, 2016(u+b); Skalny, 2021	**2 studies:** Bornstein, 1990 (only in plasma); Baker, 1991 (only in plasma, only Phe)
**Hydroxyproline**	**1 study:** Skalny, 2021		
**L-cystine**	**1 study:** Wang, 2021b		

**Ammonia, lactate**	**1 study:** Hasan, 2016		
**Kynurenine pathway**			
**Trp**	**3 studies:** Dolina, 2014(u); Evangelisti, 2017; Hoshino, 1985 (only free Trp)	**4 studies:** Bergwerff, 2016(u+b); Molina-Carballo, 2021(u+b); Sağlam, 2021; Skalny, 2021	**1 study:** Bornstein, 1990
**KYN**	**2 studies:** Evangelisti, 2017; Sağlam, 2021	**3 studies:** Kilany, 2022 (compared LD + ADHD vs. LD); Molina-Carballo, 2021(u+b); Oades, 2010a	
**3-OH-KYN**	**1 study:** Dolina, 2014(u)		**2 studies:** Oades, 2010a; Sağlam, 2021
**AA**		**1 study:** Molina-Carballo, 2021(u+b)	**1 study:** Evangelisti, 2017
**KA**	**1 study:** Dolina, 2014 (urine)	**1 study:** Sağlam, 2021	**1 study:** Evangelisti, 2017
**QA**		**2 studies:** Evangelisti, 2017; Molina-Carballo, 2021(u+b)	
**Xanthurenic acid**		**1 study:** Molina-Carballo, 2021(u+b)	**1 study:** Evangelisti, 2017
**Neurotransmitters metabolism**			
**MHPG**	**1 study:** Khan, 1981(u)	**2 studies:** Baker, 1993(u); Oades, 1998(u)	**1 study:** Shekim, 1987(u)
**DOPEG**			**1 study:** Hanna, 1996(u)
**Metanephrine, NME**	**1 study:** Konrad, 2003(u) (only NME)	**2 studies:** Baker, 1993(u); Khan, 1981(u)	
**Homovanillic acid**		**1 study:** Oades, 1998(u)	**1 study:** Shekim, 1987(u)
**5-HIAA**	**1 study:** Oades,1998(u)	**1 study:** Oades, 2010a	**2 studies:** Chatterjee, 2022; Moriarty, 2011(u)
**TIQ**	**1 study:** Roessner, 2007(u)		
**Other metabolic processes**			
**6-OH-MS**	**1 study:** Büber, 2016(u)		
**6-S-aMT**		**1 study:** Molina-Carballo, 2013(u)	
**Indolamines**		**1 study:** Fernández-López, 2020(u+b)	
**Agmatine**	**1 study:** Sari, 2020		

(u) – urine levels; (u+b) – both urine and blood levels; if not specified, the reported results reffer to levels in serum/plasma/RBCs
